# Energy-Efficient Aerial STAR-RIS-Aided Computing Offloading and Content Caching for Wireless Sensor Networks

**DOI:** 10.3390/s25020393

**Published:** 2025-01-10

**Authors:** Xiaoping Yang, Quanzeng Wang, Bin Yang, Xiaofang Cao

**Affiliations:** 1 College of Computer Science, Beijing University of Technology, Beijing 100124, China; quanzeng_w@emails.bjut.edu.cn (Q.W.); 18811419186@163.com (B.Y.); 2 School of Business, Beijing Wuzi University, Beijing 101149, China

**Keywords:** unmanned aerial vehicle, wireless sensor networks, simultaneously transmitting and reflecting reconfigurable intelligent surface, computing offloading, content caching

## Abstract

Unmanned aerial vehicle (UAV)-based wireless sensor networks (WSNs) hold great promise for supporting ground-based sensors due to the mobility of UAVs and the ease of establishing line-of-sight links. UAV-based WSNs equipped with mobile edge computing (MEC) servers effectively mitigate challenges associated with long-distance transmission and the limited coverage of edge base stations (BSs), emerging as a powerful paradigm for both communication and computing services. Furthermore, incorporating simultaneously transmitting and reflecting reconfigurable intelligent surfaces (STAR-RISs) as passive relays significantly enhances the propagation environment and service quality of UAV-based WSNs. However, most existing studies place STAR-RISs in fixed positions, ignoring the flexibility of STAR-RISs. Some other studies equip UAVs with STAR-RISs, and UAVs act as flight carriers, ignoring the computing and caching capabilities of UAVs. To address these limitations, we propose an energy-efficient aerial STAR-RIS-aided computing offloading and content caching framework, where we formulate an energy consumption minimization problem to jointly optimize content caching decisions, computing offloading decisions, UAV hovering positions, and STAR-RIS passive beamforming. Given the non-convex nature of this problem, we decompose it into a content caching decision subproblem, a computing offloading decision subproblem, a hovering position subproblem, and a STAR-RIS resource allocation subproblem. We propose a deep reinforcement learning (DRL)–successive convex approximation (SCA) combined algorithm to iteratively achieve near-optimal solutions with low complexity. The numerical results demonstrate that the proposed framework effectively utilizes resources in UAV-based WSNs and significantly reduces overall system energy consumption.

## 1. Introduction

Wireless sensor networks (WSNs), comprising a large number of sensor nodes, show impressive capability in transmitting a very large number of data with high efficiency [1,2]. Their compactness, cost-effectiveness, and ease of deployment make WSNs highly effective for a wide range of real-time applications. With the rapid development of WSNs, the explosive growth of sensor devices has intensified the demand for high data rates and ultra-low-latency services [3]. Traditional cloud computing paradigms face challenges in meeting the diverse service requirements of delay-sensitive and computing-intensive tasks [4]. Recently, mobile edge computing (MEC), which provides computing and caching services for nearby sensors, has enabled local task offloading, avoiding the need to send data to distant cloud centers and thus reducing costs [5]. This has facilitated the transition of WSN devices to large-scale deployment, allowing for the real-time monitoring of environmental conditions to offer essential insights for urban planning and management. Moreover, by deploying MEC servers at access points (APs) or base stations (BSs), it is possible not only to cache popular content on the edge cloud to reduce the delay and energy consumption of sensor content requests but also to offload tasks requiring computation [6]. However, traditional MEC servers, typically deployed at fixed base stations, often suffer from limited coverage and challenges like non-line-of-sight (NLoS) transmission, which degrades signal quality and results in reduced overall service efficiency and performance [7].

In recent years, unmanned aerial vehicles (UAVs) have gained widespread application across various industries due to their mobility, low operational cost, and ability to establish easy line-of-sight (LoS) communication links [8]. These unique characteristics enable UAVs to effectively address challenges inherent to traditional communication systems, such as fixed deployment locations, high infrastructure costs, and limited adaptability to specialized scenarios [9]. Furthermore, equipped with MEC servers, UAVs can not only provide computing and caching services to sensors but also function as aerial relays to offload tasks to other nodes, thereby significantly enhancing the flexibility and efficiency of network services [10,11,12]. However, UAVs have limited computing, caching, and endurance capabilities, thus low-power solutions are crucial to improving UAV network performance [13].

Recently presented, a promising approach to reducing UAV energy consumption involves the deployment of simultaneously transmitting and reflecting reconfigurable intelligent surfaces (STAR-RISs) as an alternative to UAVs for signal relaying [14]. Each element of a STAR-RIS, capable of supporting both electric and magnetic currents, can simultaneously reconfigure transmitted and reflected signals, thereby achieving full-space coverage [15,16]. However, most existing studies in this field assume that STAR-RISs are deployed in a fixed position [17], with UAVs primarily offering computing [18,19] or caching capabilities [20,21,22]. The fixed deployment of STAR-RISs limits their ability to flexibly adjust the distance between themselves and the sensors, thereby degrading task offloading transmission performance. Other studies have proposed mounting STAR-RISs on UAVs, but UAVs do not have computing and caching resources [23,24]. Consequently, UAVs serve only as flight carriers, leaving their potential computing and caching capabilities underutilized. This significantly reduces resource utilization and overall task processing efficiency in UAV-based WSNs.

While significant progress has been made in this field, several critical research gaps remain unaddressed. First, the fixed deployment of STAR-RISs restricts their adaptability to dynamic WSN environments, leading to suboptimal task offloading transmission performance. Second, while some studies equip UAVs with STAR-RISs, they overlook UAVs’ inherent computing and caching capabilities, resulting in the underutilization of WSN resources and reduced system efficiency. Third, existing research predominantly lacks a comprehensive joint optimization framework that simultaneously considers caching decisions, offloading strategies, UAV hovering positions, and STAR-RIS passive beamforming. These limitations hinder the ability to fully capitalize on the dynamic and heterogeneous resources of UAV-based WSNs. To address these limitations, we propose a novel STAR-RIS-assisted computing offloading and content caching framework to minimize system energy consumption for UAV-based WSNs. In this context, UAVs have computing and caching capabilities, which can greatly enhance task processing efficiency and response speed. Subsequently, given the limited energy capacity of UAVs, we optimize their energy utilization by separating the relay functionality. To this end, a STAR-RIS, as a passive relay, is introduced to assist the sensor nodes in forwarding tasks that can be reasonably allocated to UAV-based WSN resources. Furthermore, by installing a STAR-RIS on the UAV, the system can dynamically adjust the relative positioning between the STAR-RIS and the sensors, further enhancing efficiency in task transmission and processing. However, several key challenges must still be addressed to fully achieve this. First, traditional static caching strategies are not suitable for the dynamic characteristics of UAV-based WSNs. Therefore, it is essential to design effective caching strategies that ensure fast responses to sensor requests. Secondly, in order to ensure the efficient use of resources and avoid single-point overload, how to achieve the dynamic offloading of tasks among edge clouds, UAVs, and sensors is crucial. Third, due to the limited endurance of UAVs, STAR-RISs as relay nodes can effectively save the energy consumption of UAVs when forwarding tasks as relay nodes by optimizing signal transmission and reflection paths. Therefore, in order to further improve the endurance of the UAV system, it is crucial to design an effective transmission and reflection coefficient matrix for STAR-RISs. Finally, UAVs equipped with STAR-RISs not only cache and process sensor tasks but also serve as relay nodes, providing additional communication links between sensors and the edge cloud. Therefore, it is essential to jointly optimize caching decisions, offloading decisions, UAV hovering positions, and passive beamforming to minimize overall system energy consumption.

Tackling these challenges, the main contributions of this paper can be summarized as follows:

(1) We propose a novel aerial STAR-RIS-aided computing offloading and content caching framework to minimize system energy consumption for WSNs. This framework leverages flexible deployment and caching, computing, and communication (3C) resources to offer adaptive computation and caching services. Additionally, a STAR-RIS is introduced as a passive relay to assist sensors in forwarding tasks, which can reasonably allocate UAV-based WSN resources. Lastly, by installing a STAR-RIS on a UAV, the system can flexibly adjust the position between the STAR-RIS and the sensors to improve task transmission performance.
(2) Since the energy consumption minimization problem is non-convex, we decomposed the problem into four subproblems: content caching decision, computation offloading decision, UAV hovering position, and STAR-RIS resource allocation. For the subproblem of content caching decisions, the network caching decisions are optimized by utilizing a new deep reinforcement learning (DRL) algorithm. For the other subproblems, we utilize the Karush–Kuhn–Tucker (KKT) conditions and the successive convex approximation (SCA) algorithm to iteratively solve and optimize system energy consumption.
(3) The numerical results demonstrate that the proposed STAR-RIS-aided computing offloading and content caching framework significantly reduces system energy consumption in UAV-based WSNs compared with the benchmarks, especially in scenarios with limited network resources or adverse channel conditions.

The rest of this paper is organized as follows: We first briefly review the related works of this paper in Section 2 and then give the overview and mathematical description of the system model in Section 3. In Section 4 and Section 5, the optimization algorithm and iterative solution process for the proposed model are introduced. In Section 6, we discuss the convergence and complexity of the proposed DRL-SCA algorithm. Simulation environments are presented, and the results are discussed in Section 7, followed by conclusions in Section 8.

## 2. Related Works

In this section, we present related works on computation offloading and content caching in three key aspects: MEC, UAVs, and STAR-RIS-aided UAVs. The three subsections are in a progressive relationship, from MEC to UAVs and then to STAR-RIS-aided UAVs, which helps to identify gaps in existing research and gradually demonstrates the superiority of the proposed framework.

### 2.1. Computing Offloading and Content Caching in MEC

In the context of MEC, the key element is the edge server, which provides computing resources, caching capability, and connectivity [25]. When computing tasks are offloaded to MEC nodes, content caching can effectively degrade the latency and bandwidth cost of acquiring and initializing applications [26,27]. Several studies have explored the joint optimization of content caching and computing offloading in MEC [5,19,26]. Liao et al. [5] studied a joint service caching and task offloading problem in a multi-user, multi-BS, cloud-based MEC system to optimize execution delay, energy consumption, total benefit, and task offloading rate. Yu et al. [19] proposed optimal joint service caching and task offloading strategies to minimize the overall execution delay at the mobile terminal side in a MEC scenario. Bi et al. [26] minimized user computation delay and energy consumption by jointly optimizing resource allocation, computing offloading, and content caching decisions in MEC networks.

### 2.2. Computing Offloading and Content Caching in UAVs

Although MEC can provide low-cost computing and caching services for nearby sensors, MEC is typically deployed at fixed BSs, which limits its adaptability to sensors’ demands, coverage, and service quality [5,7]. To overcome these limitations, UAV communication networks have become crucial due to their high mobility, strong LoS links, and fast deployment capabilities [28]. The UAVs equipped with MEC servers can plan their hovering positions or flight trajectories strategically, offering 3C resources dynamically [20]. Several studies have investigated the joint optimization of computing offloading and content caching in UAVs [21,22]. Zhao et al. [21] studied a UAV-enabled MEC network where UAVs equipped with caching and computation capabilities collaborate with ground BSs to fulfill sensor requests, enhancing communication resources for data transmission. Huang et al. [22] proposed a UAV-assisted Internet of Vehicles (IoV) framework, where both UAVs and BSs provide computing and caching services for smart vehicles, to minimize average task processing delay and maximize the UAV cache hit ratio.

### 2.3. Computing Offloading and Content Caching in STAR-RIS-Aided UAVs

Although UAVs can provide flexible computing and caching services, it is difficult to meet high-energy-consuming task demands due to the limited endurance capabilities of UAVs [13,17,29]. Therefore, low-power solutions are essential to improving the performance of UAV-based WSNs. Fortunately, a promising approach to reducing UAV energy consumption is the deployment of STAR-RISs as an alternative to UAVs for signal relaying [23,30]. Aung et al. [23] deployed STAR-RISs on UAVs and minimized energy consumption for IoT devices and aerial STAR-RISs by jointly optimizing task offloading, aerial STAR-RIS trajectory, amplitude, phase shift coefficients, and transmitting power in a STAR-RIS-aided MEC network. Zhang et al. [30] maximized the sum rate of all users by jointly optimizing the STAR-RISs’ beamforming vectors, the UAVs’ trajectory, and the power allocation STAR-RIS-assisted UAV communication system.

However, most studies assume that STAR-RISs are fixed and UAVs only provide computing or caching capabilities. Since STAR-RISs cannot flexibly adjust the distance between themselves and the sensor, task offloading transmission performance is reduced [30]. Some other studies assumed that STAR-RISs are mounted on UAVs but UAVs do not have computing and caching resources [23]. Since a UAV can only serve as a flight carrier, the computing and caching service resources of UAV-based WSNs cannot be utilized, which significantly reduces resource utilization and overall task processing efficiency. To overcome these limitations, we propose a novel energy-efficient aerial STAR-RIS-assisted computing offloading and content caching framework, which installs a STAR-RIS on a UAV, fully leveraging UAV-based WSNs’ computational and caching capacities for improved resource utilization and task execution efficiency. In this framework, we formulate a problem to minimize system energy consumption by jointly optimizing the content caching decisions, computing offloading decisions, UAV hovering positions, and STAR-RIS passive beamforming.

## 3. System Model and Problem Formulation

In this section, we provide a comprehensive description of the network overview, communication model, computation model, caching model, and energy consumption model for the proposed aerial STAR-RIS-aided WSN. We also formulate a system energy consumption minimization optimization problem. The notation is summarized in Table 1.

### 3.1. Network Overview

**Figure 1:**
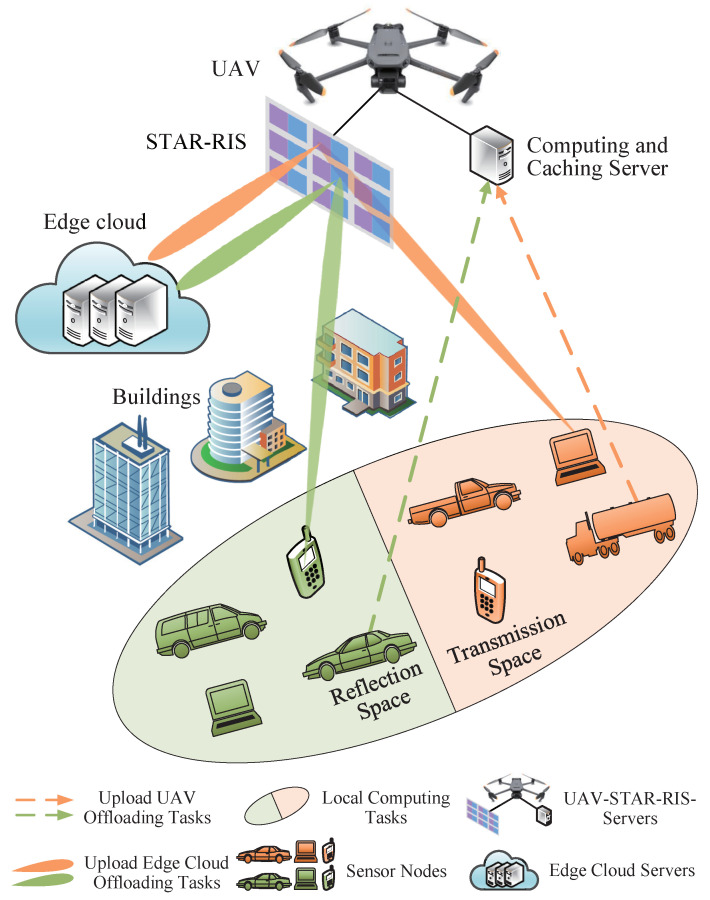
System model of aerial STAR-RIS-aided WSN.

As shown in Figure 1, the considered aerial STAR-RIS-aided WSN consists of an edge cloud *c*, a UAV *u* equipped with computing and caching servers and a STAR-RIS, and many single-antenna sensors. UAV *u* can provide services for sensors and those sensors are denoted by M={1,2,…,m,…,M}. And the set of tasks is denoted by K={1,2,…,k,…,K}. Moreover, we assume that content popularity follows the Zipf distribution. We consider that edge cloud *c* and UAV *u* have limited caching and computing capacities and that the sensors only have limited computing capacities. The UAV has mobility and can adjust its position relative to the connected sensors to improve the quality of signal transmission. In this network, UAV *u* is connected to edge cloud *c* and to the sensors by wireless links. In particular, the STAR-RIS consists of $M_{c}$ × $M_{r}$ passive units, which are spanned as a uniform planar array (UPA). Each column and row of the UPA has $M_{c}$ and $M_{r}$ passive units, respectively [31]. The $\left(m_{c},m_{r}\right)$-th STAR-RIS element is used to represent the element of the $m_{r}$-th row and $m_{c}$-th column of the STAR-RIS. We assume that the direct communication links between the sensors and edge cloud *c* are blocked and that the STAR-RIS elements work in energy-splitting (ES) mode [32]. In the proposed STAR-RIS-aided UAV system, the sensor *m* can process the partial tasks; then, UAV *u* can partially process the remaining tasks uploaded by the sensors and return the results to the sensors. At the same time, the other remaining tasks are forwarded to edge cloud *c* for processing through the STAR-RIS elements, and the results are returned to the sensors. In this paper, the main focus is on minimizing system energy consumption in the STAR-RIS-aided UAV system by jointly optimizing the content caching decisions, computing offloading decisions, UAV hovering positions, and STAR-RIS passive beamforming.

**Figure 2:**
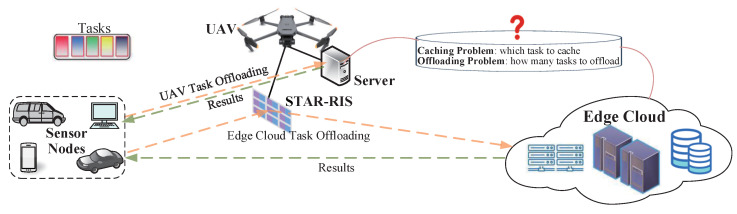
Illustration of task caching and offloading for STAR-RIS-aided UAV system.

As illustrated in Figure 2, we make use of virtual reality (VR) as a typical application scenario. The sensors need to process a variety of virtual reality application tasks, such as object tracking, object identification, and scene rendering. Every task requires a different amount of computing capacity and data size. For instance, object-tracking tasks require a large number of data to be transmitted, while object identification tasks and scene-rendering tasks require greater computing resources. Therefore, we adopt two parameters in total for modeling heterogeneous computation tasks. For computation task *k*, we define $F_{k}=\left\{\omega_{k}\;L_{k}\right\}$, where $\omega_{k}$ represents the computing resources required to complete the task and $L_{k}$ represents the data size of the task, i.e., the size of the data that need to be transmitted to UAV *u* or edge cloud *c*.

**Figure 3:**
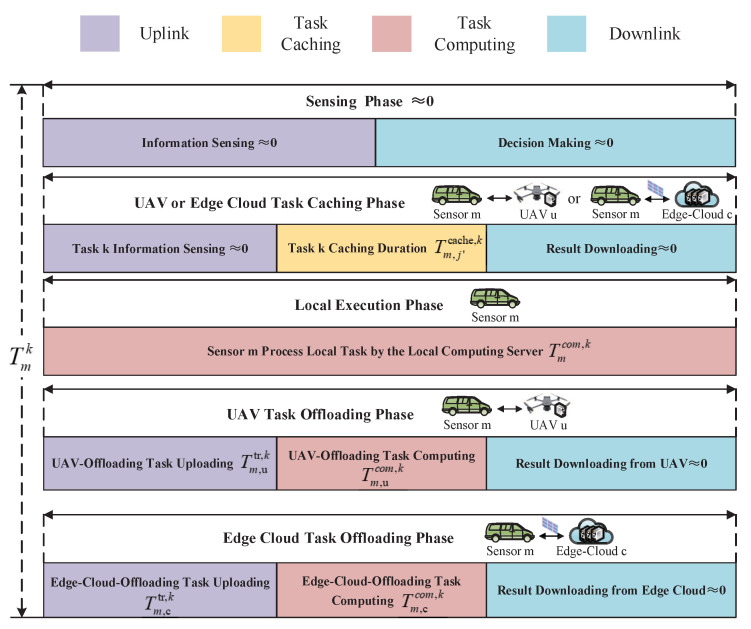
Time allocation for task processing in STAR-RIS-aided UAV system.

When the computing task is not cached in the system, we consider the partial offloading scheme for delay-sensitive computation tasks in STAR-RIS-aided UAV systems. This kind of computation offloading model allows tasks to be calculated in parallel at the sensors, UAV *u*, and edge cloud *c*. The tasks processed at the sensors are referred to as local tasks, the tasks processed at UAV *u* are referred to as UAV-offloading tasks, and the tasks that are offloaded to edge cloud *c* are called edge cloud-offloading tasks. Figure 3 presents the time allocation for task processing in the STAR-RIS-aided UAV system, where the sensors utilize the same resource block with duration $T_{m}^{k,\max}$ to transmit and compute tasks.

In the local execution phase, the sensors process their tasks by the local computing servers. In the UAV task offloading phase, some of the remaining tasks are uploaded to UAV *u* and are processed by the computing server of UAV *u*. In the edge cloud task offloading phase, some of the remaining tasks are forwarded to edge cloud *c* through the STAR-RIS elements for processing. When the tasks are completed, the computing results obtained at both UAV *u* and edge cloud *c* are returned to the sensors. In downlink communication, since UAV *u* and edge cloud *c* tend to have high transmit power and the computing results are usually of small size, the downloading time is comparatively negligible in the UAV task offloading phase and edge cloud task offloading phase.

### 3.2. Communication Model

This subsection introduces the communication model and gives the uplink data rate when the sensor offloads tasks on UAV *u* and edge cloud *c*. We assume that when the task is offloaded, the sensor does not move. A 3D Cartesian coordinate system is established to describe the locations of the sensors, UAV *u*, STAR-RIS, and edge cloud *c*. The locations of sensor *m* and edge cloud *c* are described by vector $r_{m}=\left(x_{m},y_{m},0\right)$ and vector $r_{c}=\left(x_{c},y_{c},0\right)$. We assume that UAV *u* is hovering at a fixed position in every slot to provide computing services for the sensors [33]. The position of UAV *u* is $r_{u}=\left(x_{u},y_{u},h_{u}\right)$, and the position of the $\left(m_{c},m_{r}\right)$-th STAR-RIS element is $r_{\left(m_{c},m_{r}\right)}=\left(x_{\left(m_{c},m_{r}\right)},y_{\left(m_{c},m_{r}\right)},h_{\left(m_{c},m_{r}\right)}\right)$.

Due to the high probability of LoS links in UAV communication, the communication channels between sensors *m* and UAV *u*, between sensor *m* and the $\left(m_{c},m_{r}\right)$-th STAR-RIS element, and between the $\left(m_{c},m_{r}\right)$-th STAR-RIS element and edge cloud *c* are assumed to be LoS links, all following the free-space path loss model [34]. So, the channel gain between node *n* and another node $n^{\prime }$ can be formulated as follows:

(1) $$\begin{array}{cc} h_{n,n^{\prime }}= & \sqrt{g_{0}}d_{n,n^{\prime }}^{-2}, \end{array}$$

where $n\neq n^{\prime }$, $n\in \left\{m,(m_{c},m_{r})\right\}$, and $n^{\prime }\in \left\{u,(m_{c},m_{r}),c\right\}$. $g_{0}$ is the received power at a distance of 1 *m* for a transmission power of 1 *W* and $d_{n,n^{\prime }}=\sqrt{{\left(x_{n}-x_{n^{\prime }}\right)}^{2}+{\left(y_{n}-y_{n^{\prime }}\right)}^{2}+{\left(h_{n}-h_{n^{\prime }}\right)}^{2}}$. The signal-to-interference-plus-noise ratio (SINR) of the wireless link from node *n* to another node $n^{\prime }$, denoted by $\gamma_{n,n^{\prime }}$, can be expressed as

(2) $$\gamma_{n,n^{\prime }}=\frac{P_{n}{\left|h_{n,n^{\prime }}\right|}^{2}}{\sigma_{n,n^{\prime }}^{2}},$$

where $P_{n}$ is the transmit power of node *n*. We assume that the noise power has constant variance $\sigma_{n,n^{\prime }}^{2}$. Therefore, the transmission rate from node *n* to node $n^{\prime }$ can be given by

(3) $$R_{n,n^{\prime }}=B_{n,n^{\prime }}\log_{2}\left(1+\frac{P_{n}{\left|h_{n,n^{\prime }}\right|}^{2}}{\sigma_{n,n^{\prime }}^{2}}\right),$$

where $B_{n,n^{\prime }}$ is the transmission bandwidth of the wireless link from node *n* to node $n^{\prime }$.

#### 3.2.1. Channel in UAV Task Offloading

UAV *u* equipped with the MEC server has computing resources, which allows the sensors to transfer some tasks to UAV *u* for processing. The signal received by UAV *u* can be written as

(4) $$z_{u}=\sum_{m\in M}h_{m,u}\sqrt{P_{m}}s_{m}+n_{u},$$

where $s_{m}$ is the corresponding signal with $E\left\{|s_{m}{|}^{2}\right\}=1$ [35]. $n_{u}$ is the additive white Gaussian noise (AWGN) received by UAV *u*. The noise power is $\sigma_{u}^{2}$, i.e., $n_{u}=n∼N\left(0,\sigma_{u}^{2}\right)$.

According to (2) and (3), the SNR and transmission rate of the link from sensor *m* to UAV *u* are denoted by $\gamma_{m,u}$ and $R_{m,u}$.

#### 3.2.2. Channel in Edge Cloud Task Offloading

Subject to its energy limitations and task time constraints, UAV *u* can only perform part of its received remaining tasks. The remaining tasks are forwarded to edge cloud *c* for processing through the STAR-RIS. In order to better distinguish the channel gain from sensor *m* to the $\left(m_{c},m_{r}\right)$-th STAR-RIS element, the channel gain from the $\left(m_{c},m_{r}\right)$-th STAR-RIS element to edge cloud *c* is expressed as $g_{(m_{c},m_{r}),c}$. Therefore, the channel gains from sensor *m* to the STAR-RIS and from the STAR-RIS to edge cloud *c* are denoted by $H_{m,s}\in C^{M_{c}M_{r}\times 1}$ and $G_{s,c}^{H}\in C^{1\times M_{c}M_{r}}$, respectively. $\Theta_{a}=\mathrm{diag}\left[\sqrt{\beta_{\left(1,1\right)}^{a}}e^{j\theta_{\left(1,1\right)}^{a}},\sqrt{\beta_{\left(1,2\right)}^{a}}e^{j\theta_{\left(1,2\right)}^{a}},\ldots ,\sqrt{\beta_{\left(m_{c},m_{r}\right)}^{a}}e^{j\theta_{\left(m_{c},m_{r}\right)}^{a}},\ldots ,\sqrt{\beta_{\left(M_{c},M_{r}\right)}^{a}}e^{j\theta_{\left(M_{c},M_{r}\right)}^{a}}\right]$ is the transmission or reflection coefficient matrix of the STAR-RIS for the incident signal from sensor *m*, where $\sqrt{\beta_{\left(m_{c},m_{r}\right)}^{a}}\in \left[0,1\right]$ and $\theta_{\left(m_{c},m_{r}\right)}^{a}\in \left[0,2\pi \right)$ denote the amplitude and phase shift of the $\left(m_{c},m_{r}\right)$-th STAR-RIS element for the sensor’s signal, respectively, and $a\in \left\{r,t\right\}$. Let $\Theta_{a}$ be the reflection (a=r) or transmission (a=t) beamforming vectors. In consequence, the following constraint is required when the STAR-RIS is in ES mode:

(5) $$\sum_{a\in \left\{r,t\right\}}\beta_{\left(m_{c},m_{r}\right)}^{a}\leq 1.$$

Hence, the signal received at edge cloud *c* can be obtained as

(6) $$z_{c}=\sum_{m\in M}G_{s,c}^{H}\Theta_{a}H_{m,s}\sqrt{P_{m}}s_{m}+n_{c},$$

where $n_{c}$ is the AWGN received at edge cloud *c* with variance $\sigma_{c}^{2}$, i.e., $n_{c}=n∼N\left(0,\sigma_{c}^{2}\right)$. If sensor *m* is in the transmission region, then a=t; otherwise, if sensor *m* is in the reflection region, a=r.

Similarly, the SNR and transmission rate of the link from sensor *m* to edge cloud *c* via the STAR-RIS are denoted by $\gamma_{m,c}$ and $R_{m,c}$, respectively, where the channel gain from sensor *m* via the STAR-RIS to edge cloud *c* is $G_{s,c}^{H}\Theta_{a}H_{m,s}$.

We do not take packet loss and downlink transmission delay into account in this paper. This is due to the fact that the downlink transition rate is higher than the uplink transition rate and the size of the data after task processing is much smaller than that of the data before processing.

### 3.3. Computation Model

In this study, we consider a divisible computation task, allowing it to be segmented into multiple parts. Taking video analysis as an example, a large video file containing numerous frames can be divided into several video clips through segmentation. This enables a portion of the clips to be initially processed locally at the sensor, while others are handled by UAV *u*, and the remaining clips are offloaded to edge cloud *c*. Detailed explanations will be provided in subsequent sections. Additionally, we disregard the delay associated with transmitting the processed task results from the UAV and edge cloud back to the sensors, as the output data size is significantly smaller compared with that of the input data.

We define the integer caching decision variable, $X_{j^{\prime }}^{k}\in \left\{0,1\right\}$$\left(j^{\prime }\in \left\{u,c\right\}\right)$, which indicates whether task k is cached at node $j^{\prime }$ ($X_{j^{\prime }}^{k}$ = 1) or not ($X_{j^{\prime }}^{k}$ = 0). Therefore, the task caching strategy can be represented as follows: $X=\left\{X_{j^{\prime }}^{1},X_{j^{\prime }}^{2},\ldots ,X_{j^{\prime }}^{k}\right\}$. For the task k offloading problem of sensor m, we define decision variable $\alpha_{m,j}^{k}\in \left[0,1\right]$, j∈{m,u,c}. $\alpha_{m,j}^{k}\in \left[0,1\right]$ is the task offloading ratio of sensor *m* at node *j*. (In this paper, we assume that sensor *m* initiates a task request to node *j*. Node *j* senses the information of the task (task size and time constraints) and then makes a task offloading decision based on its own computing capability. We call the above process the sensing phase, and since this time is very short, we ignore this time.) Consequently, the following is a representation of the task offloading policy: $\alpha =\left\{\alpha_{m,m}^{k},\alpha_{m,u}^{k},\alpha_{m,c}^{k}\right\}$, $\sum_{j\in \{m,u,c\}}\alpha_{m,j}^{k}=1$.

Service delay is the overall service time for task *k* when task k is not cached, consisting of two parts: (i) uplink communication delay; (ii) computation delay. In downlink communication, since the UAV and edge cloud tend to have high transmit power and the computing results are usually of small size, the downloading time is also negligible. Next, we discuss the delay and corresponding energy consumption.

#### 3.3.1. Energy Consumption for Uplink Communication

We denote $X_{j^{\prime }}^{k}=0$, as task k is not cached, and offloading decision variable $\alpha_{m,j^{\prime }}^{k}$. Therefore, the uplink transmission delay of offloading task k from sensor *m* to node $j^{\prime }$ can be expressed as

(7) $$T_{m,j^{\prime }}^{tr,k}=\frac{\alpha_{m,j^{\prime }}^{k}L_{k}}{R_{m,j^{\prime }}}.$$

Therefore, when task k is not cached, the total uplink communication energy consumption for sensor *m* offloading task k can be calculated as

(8) $$\begin{array}{cc} E_{m}^{tr,k}= & \sum_{j^{\prime }\in \left\{u,c\right\}}P_{m}T_{m,j^{\prime }}^{tr,k}. \end{array}$$

#### 3.3.2. Energy Consumption for Computation

We denote $X_{j^{\prime }}^{k}=0$, as task k is not cached, and offloading decision variable $\alpha_{m,j}^{k}$. Therefore, the computation delay for computing offloading task *k* at node *j* can be expressed as

(9) $$\begin{array}{c} T_{m,j}^{com,k}=\frac{\alpha_{m,j}^{k}\omega_{k}}{f_{j}}, \end{array}$$

where $\omega_{k}$ is the number of required computation resources for task *k*, i.e., the number of CPU cycles required for computing 1-bit task data. $f_{j}$ is the computing capability (CPU cycles per second) of node *j*.

Therefore, the total computing energy consumption of sensor *m* for computing task request *k* can be expressed as

(10) $$\begin{array}{cc} E_{m}^{com,k} & =\sum_{j\in \{m,u,c\}}E_{m,j}^{com,k}\\ \; & =\sum_{j\in \{m,u,c\}}\kappa_{j}T_{m,j}^{com,k}f_{j}^{3}, \end{array}$$

where $\kappa_{j}$ is the effective capacitance coefficient of node *j* that depends on the processor’s chip architecture.

### 3.4. Caching Model

In this subsection, we describe the caching model. Task caching involves storing completed tasks and associated data within UAV *u* or edge cloud *c*. Specifically, an independent resource container is maintained on UAV *u* or edge cloud *c*. The caching process operates as follows: Firstly, sensors send a computing task request. If UAV *u* or edge cloud *c* has already cached this task, the respective node notifies the sensors of its availability on the caching servers. Consequently, the sensor can avoid offloading the same task to UAV *u* or edge cloud *c*. At last, after the task is processed by UAV *u* or edge cloud *c*, the results are sent back to the sensors. This caching mechanism reduces the need for redundant task offloading, thereby lowering sensor energy consumption and minimizing offloading delays.

Despite its benefits, task caching still faces many challenges: (i) although UAV *u* and edge cloud *c* have greater caching and computational capacities compared with sensors, they still cannot cache or handle all kinds of computation tasks; (ii) unlike traditional caching strategies, task caching requires the consideration of not only the data size and computational resources necessary for each task but also task popularity. Consequently, designing an effective caching strategy presents significant challenges. We introduce an integer caching decision variable, $X_{j^{\prime }}^{k}\in \left\{0,1\right\}$, to indicate whether task *k* is cached at node $j^{\prime }$ ($X_{j^{\prime }}^{k}$ = 1) or not ($X_{j^{\prime }}^{k}$ = 0). Therefore, the computation caching strategy can be defined as $X=\left\{X_{j^{\prime }}^{1},X_{j^{\prime }}^{2},\ldots ,X_{j^{\prime }}^{k}\right\}$. In this study, we evaluate the task duration and energy consumption on UAV *u* or edge cloud *c* in scenarios with and without task caching. For task caching ($X_{j^{\prime }}^{k}$ = 1), the task duration, simplified to the processing delay, is denoted by $T_{m,j^{\prime }}^{cache,k}$ and can be expressed as

(11) $$T_{m,j^{\prime }}^{cache,k}=\frac{\omega_{k}}{f_{j^{\prime }}}.$$

The primary energy consumption occurs within UAV *u* or edge cloud *c*, with sensors incurring no energy cost. Accordingly, the energy consumption associated with task caching can be formulated as

(12) $$\begin{array}{cc} E_{m,j^{\prime }}^{cache,k}= & \kappa_{j^{\prime }}T_{m,j^{\prime }}^{cache,k}f_{j^{\prime }}^{3},\; \end{array}$$

where $\kappa_{j^{\prime }}$ is the effective capacitance coefficient of node $j^{\prime }$ that depends on the processor’s chip architecture.

### 3.5. Problem Formulation

Based on the task communication, computation, and caching process mentioned above, the overall delay and energy consumption of sensor *m* from sending task request *k* to obtaining computing results are expressed as

(13) $$\begin{array}{cc} T_{m}^{k} & =\sum_{j^{\prime }\in \left\{u,c\right\}}X_{j^{\prime }}^{k}T_{m,j^{\prime }}^{cache,k}+\left(1-\sum_{j^{\prime }\in \left\{u,c\right\}}X_{j^{\prime }}^{k}\right)\max\left\{\kappa_{m}T_{m,m}^{com,k}f_{m}^{3},\max_{j^{\prime }\in \left\{u,c\right\}}\left\{T_{m,j^{\prime }}^{tr,k}+T_{m,j^{\prime }}^{com,k}\right\}\right\};\; \end{array}$$

(14) $$\begin{array}{cc} E_{m}^{k} & =\sum_{j^{\prime }\in \left\{u,c\right\}}X_{j^{\prime }}^{k}E_{m,j^{\prime }}^{cache,k}+\left(1-\sum_{j^{\prime }\in \left\{u,c\right\}}X_{j^{\prime }}^{k}\right)\left(E_{m}^{\mathrm{tr},\;k}+E_{m}^{com,k}\right).\; \end{array}$$

In this paper, for task caching ($X_{j^{\prime }}^{k}$ = 1), the primary energy consumption occurs within UAV *u* or edge cloud *c*, with sensors incurring no energy cost. The primary energy consumption, simplified to the processing energy consumption, is denoted by $E_{m,j^{\prime }}^{cache,k}$. For task caching ($X_{j^{\prime }}^{k}$ = 0), the primary energy consumption includes the total uplink communication energy consumption for sensor *m* offloading task *k* and the total computing energy consumption of computing task request *k*. Therefore, we formulate a system energy consumption minimization problem by jointly optimizing caching decision $X=\left\{X_{j^{\prime }}^{1},X_{j^{\prime }}^{2},\ldots ,X_{j^{\prime }}^{k}\right\}$ $\left(j^{\prime }\in \left\{u,c\right\}\right)$, offloading decision $\alpha_{m,j}^{k}\in \left[0,1\right]$ (j∈{m,u,c}), hovering position of UAV *u* $r_{u}$, and passive beamforming Θ in the STAR-RIS-aided UAV system. According to (14), the optimization problem for minimizing the total energy consumption of the STAR-RIS-aided UAV system can be formulated as

(15) $$\begin{array}{cc} P_{1}:\;\; & \min_{\begin{array}{c} X,\alpha ,r_{u},\Theta \end{array}}\sum_{m\in M}\sum_{k\in K}E_{m}^{k} \end{array}$$

(15a) $$\begin{array}{cc} s.t.\;\; & C_{1}:\alpha_{m,j}^{k}\in \left[0,1\right],\forall m,j\in \left\{m,u,c\right\}, \end{array}$$

(15b) $$\begin{array}{cc} \; & C_{2}:\sum_{j\in \left\{m,u,c\right\}}\alpha_{m,j}^{k}=1, \end{array}$$

(15c) $$\begin{array}{cc} \; & C_{3}:\sum_{a\in \left\{r,t\right\}}\beta_{\left(m_{c},m_{r}\right)}^{a}\leq 1,\beta_{\left(m_{c},m_{r}\right)}^{a}\in \left[0,1\right],\forall a,\forall m_{c},\forall m_{r}, \end{array}$$

(15d) $$\begin{array}{cc} \; & C_{4}:\theta_{\left(m_{c},m_{r}\right)}^{a}\in \left[0,2\pi \right),\forall a,\forall m_{c},\forall m_{r}, \end{array}$$

(15e) $$\begin{array}{cc} \; & C_{5}:x_{u}\leq x_{u}^{\max},y_{u}\leq y_{u}^{max}, \end{array}$$

(15f) $$\begin{array}{cc} \; & C_{6}:\sum_{k\in K}X_{j^{\prime }}^{k}S^{k}\leq O_{j^{\prime }},\forall k,\forall j^{\prime }, \end{array}$$

(15g) $$\begin{array}{cc} \; & C_{7}:\sum_{k\in K}\alpha_{m,j}^{k}\omega_{k}\leq f_{j},\forall k,\forall m,\forall j, \end{array}$$

(15h) $$\begin{array}{cc} \; & C_{8}:X_{j^{\prime }}^{k}\in \left\{0,1\right\},\forall k,\forall j^{\prime }, \end{array}$$

(15i) $$\begin{array}{cc} \; & C_{9}:T_{m}^{k}\leq T_{m}^{k,\max},\forall m,\forall k. \end{array}$$

Constraint $C_{1}$ ensures that the task offloading ratio at each sensor must be between 0 (indicating no offloading) and 1 (indicating full offloading), ensuring that the offloading task is appropriately distributed. Constraint $C_{2}$ ensures that the sum of offloading ratios for task *k* across all offloading targets (sensors *m*, UAV *u*, and edge cloud *c*) is equal to 1. Constraint $C_{3}$ ensures that it governs the amplitude response of the $\left(m_{c},m_{r}\right)$-th STAR-RIS element, limiting it to a range of [0,1]. Constraint $C_{4}$ defines the phase shift of the $\left(m_{c},m_{r}\right)$-th STAR-RIS element, ensuring that it lies within the range [0,2π). This is because the phase shift is a periodic quantity, and its effective range should be between 0 and 2π. Constraint $C_{5}$ restricts the UAV’s coordinates, $x_{u}$ and $y_{u}$, which must not exceed the maximum allowed values $x_{u}^{\max}$ and $y_{u}^{\max}$, ensuring that the UAV operates within a defined area. Constraint $C_{6}$ ensures that the total cached content at each node does not exceed its maximum caching capacity $O_{j^{\prime }}$. Constraint $C_{7}$ ensures that the total computational resources allocated to handle tasks at each node do not exceed its maximum computation capacity $f_{j}$. Constraint $C_{8}$ restricts the caching binary decision variables $X_{j^{\prime }}^{k}$ to take the value of either 0 or 1. This is typically used to represent whether a task is cached to a specific node or not. Constraint $C_{9}$ ensures that the completion time of task *k* does not exceed the maximum tolerable time $T_{m}^{k,\max}$ for the task. This guarantees that all tasks are completed within the allowed time window, ensuring that delay requirements are met.

Due to the different hovering positions of UAV *u*, the channel gain of the sensor-to-UAV channel link and the sensor-to-STAR-RIS-to-edge cloud channel link may differ, which in turn affects the transmission energy consumption. For ease of calculation, we assume that the $h_{u}$ of UAV *u* is fixed [36]. We optimize the $x_{u}$ and $y_{u}$ of UAV *u* to minimize the total energy consumption of the system. Meanwhile, we assume that the coordinates of STAR-RIS are the same as those of UAV *u*.

**Figure 4:**
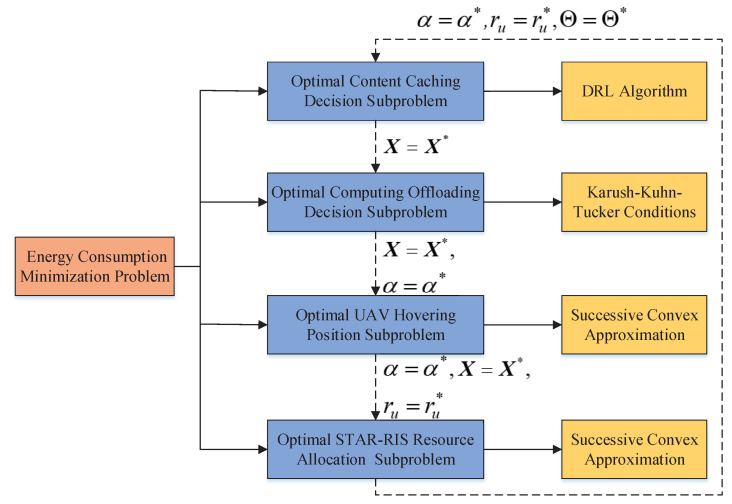
The proposed optimization framework of the energy consumption minimization problem.

As shown in Figure 4, we adopt alternative optimization techniques and decompose problem (15) into four subproblems:

(1) *Content caching decision subproblem:* Given $\alpha =\alpha^{0}$, $r_{u}=r_{u}^{0}$, and $\Theta =\Theta^{0}$, i.e., when $\alpha ,r_{u},$ and Θ are fixed, problem (15) optimizes the caching decision vector X to minimize the total energy consumption of the system. We adopt the DRL algorithm to optimize the content caching decision, denoted by $X^{*}$.
(2) *Computing offloading decision subproblem:* Given $X=X^{*}$, $r_{u}=r_{u}^{0}$, and $\Theta =\Theta^{0}$, i.e., when $X,r_{u},$ and Θ are fixed, problem (15) optimizes the offloading decision vector α to minimize the total energy consumption of the system. We adopt the KKT conditions to obtain an optimal solution, denoted by $\alpha^{*}$.
(3) *UAV hovering position subproblem:* Given $X=X^{*}$, $\alpha =\alpha^{*}$, and $\Theta =\Theta^{0}$, i.e., when α,X, and Θ are fixed, problem (15) optimizes the hovering position of UAV *u* vector $r_{u}$ to minimize the total energy consumption of the system. We adopt the SCA method to optimize the hovering position of UAV *u*, denoted by $r_{u}^{*}$.
(4) *STAR-RIS resource allocation subproblem:* Given $X=X^{*}$, $\alpha =\alpha^{*}$, and $r_{u}=r_{u}^{*}$, the transmission and reflection coefficient matrix Θ is optimized to minimize the total energy consumption of the system. We adopt the SCA method to obtain an optimal solution, denoted by $\Theta^{*}$.

## 4. Content Caching Decision Optimization

Since content caching decision optimization is only related to X but independent of other variables in $P_{1}$, caching decision optimization can be solved in advance with given offloading decisions and the hovering position of the UAV, as well as the transmission and reflection coefficient matrix. The subproblem can be written as

(16) $$\begin{array}{cc} P_{2}:\;\; & \min_{\begin{array}{c} X \end{array}}\sum_{m\in M}\sum_{k\in K}E_{m}^{k}\\ \; & s.t.\;\left(C_{6}\right)-\left(C_{9}\right) \end{array}$$

Due to the fact that X is a binary vector, $P_{2}$ is still subject to a mixed-integer nonlinear programming (MINLP) problem. Traditional optimization methods like SCA may not effectively handle such problems, especially in environments where the network state changes rapidly and unpredictably. The traditional binary-relax SCA approach first relaxes the binary variables X from the discrete space {0,1} into the continuous space [0, 1] and then forces them to round back after the SCA-based iterations. Therefore, such “relaxation” may lead the solution to converge to a local minimum in real-time dynamic systems.

**Figure 5:**
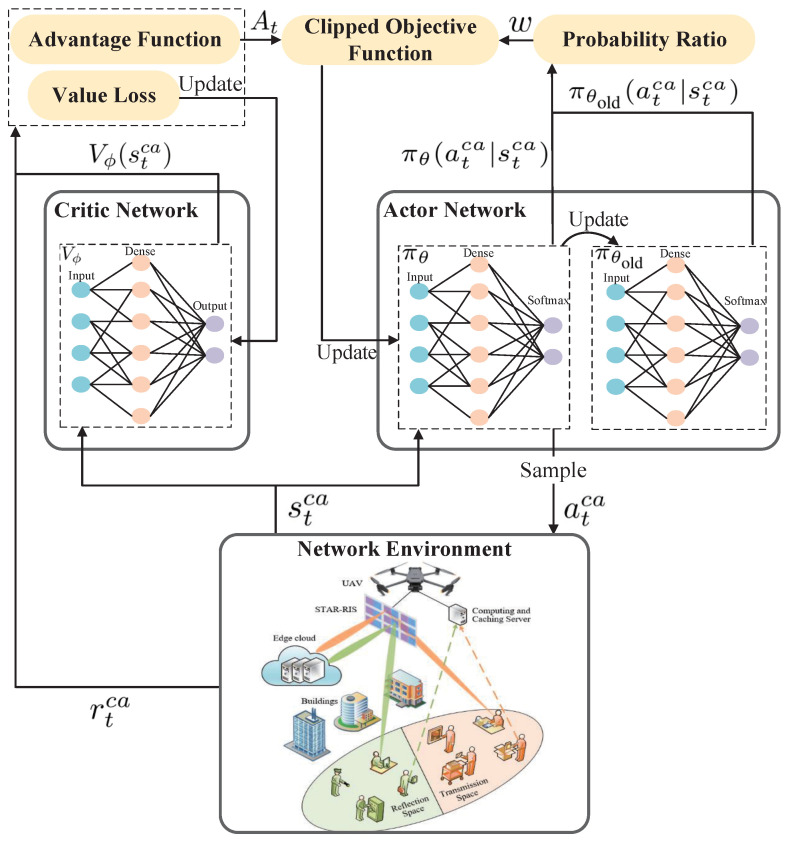
Workflow of PPO algorithm.

To address these challenges, we utilize the proximal policy optimization (PPO) algorithm, a DRL method. As shown in Figure 5, the PPO algorithm uses neural networks to model complex relationships between system states and actions, learning directly from interactions with the environment. By leveraging the PPO algorithm, caching decisions are dynamically adjusted based on real-time network conditions, such as caching state, content popularity, historical request access frequency, and network topology. The intelligent agent iteratively updates its caching strategy to maximize a reward function reflecting caching efficiency, like the cache hit rate, enabling near-optimal caching strategies that adapt to changing conditions and enhance overall system performance.

PPO is implemented within the actor–critic framework, comprising a policy network (actor) and a value network (critic). In this setup, the actor generates actions, while the critic evaluates them. A significant limitation of the basic actor–critic framework is its low sample efficiency, which requires extensive interactions with the environment to converge. To address this, PPO introduces two major contributions: mini-batch updates to improve data efficiency and a clipped surrogate loss to constrain policy updates. The PPO algorithm allows for a small difference between the target policy $\pi_{\theta }\left(a_{t}^{ca}|s_{t}^{ca}\right)$ and the behavior policy $\pi_{\theta_{\mathrm{old}}}\left(a_{t}^{ca}|s_{t}^{ca}\right)$, where $a_{t}$ and $s_{t}$ denote the action taken and the state observed at time *t*. This is achieved by using a clipping function that limits the extent of policy change. If the policy update exceeds a predefined threshold, the clipping function prevents further increase.

### 4.1. Intelligent Caching MDP Model

In the content caching decision subproblem for aerial STAR-RIS-aided WSNs, we leverage caching state, content popularity, historical request frequency, and network topology data to realize the optimal content caching decisions. The caching update model is formulated as a Markov decision process (MDP). In each time slot t∈T, the agent observes the current state, $s_{t}^{ca}\in S^{ca}$, and selects an action $a_{t}^{ca}\in A^{ca}$. Upon executing action $a_{t}^{ca}$, the agent receives an immediate reward $r_{t}^{ca}$ and the environment transitions to the next state, $s_{t+1}^{ex}$. The transition tuple $\left(s_{t}^{ca},a_{t}^{ca},r_{t}^{ca},s_{t+1}^{ca}\right)$ is stored in an experience replay buffer for agent training. To derive the optimal solution for problem $P_{2}$ under heterogeneous scenarios, state space $s_{t}^{ca}$, action space $a_{t}^{ca}$, and reward function $r_{t}^{ca}$ in the proposed intelligent caching MDP model are designed as follows:

(1) State: State $s_{t}^{ca}$ in slot t includes caching state information $M_{t}$, content popularity $P_{t}$, historical request access frequency $F_{t}$, and network topology information $G_{t}$. Thus, the state vector in slot t is expressed as
  (17) $$\begin{array}{c} s_{t}^{ca}=\left\{M_{t},P_{t},F_{t},G_{t}\right\}, \end{array}$$
  where $M_{t}$ represents the content caching status across all caching nodes in time slot *t*, expressed as $M_{t}=\left\{M_{u,t},M_{c,t}\right\}$. Additionally, $F_{t}=\left\{F_{1,t},\ldots ,F_{k,t},\ldots ,F_{K,t}\right\}$ denotes the historical access frequencies for all requests.
(2) Action: During the process of caching decision, the optimal content caching decision, $a_{t}^{ca}$, includes the cached content across all nodes for the upcoming time slot t+1. The expression for $a_{t}^{ca}$ is given by
  (18) $$\begin{array}{cc} a_{t}^{ca}= & [C_{1,c,t+1},\ldots ,C_{k,c,t+1},\ldots ,C_{K,c,t+1},C_{1,u,t+1},\ldots ,C_{k,u,t+1},\ldots ,C_{K,u,t+1}], \end{array}$$
  where $C_{k,c,t+1}$ represents whether the content of task request *k* is cached in edge cloud *c* in time slot t + 1.
(3) Reward: The formulation of the reward function plays a crucial role in guiding the exploration of the caching update problem and ensuring algorithm convergence. Consequently, the reward function in time slot *t* is defined as follows:
  (19) $$\begin{array}{c} r_{t}=\lambda \sum_{k=1}^{N_{st}}R_{k}+\left(1-\lambda \right)\sum_{j^{\prime }\in \left\{u,c\right\}}H_{t}^{j^{\prime }}, \end{array}$$
  where $N_{st}$ is the maximum training steps, λ is the weights parameter, $H_{t}^{j^{\prime }}$ is the number of cache hits for node $j^{\prime }$ in time slot t, and $R_{k}$ represents sensor satisfaction at step k.

### 4.2. PPO-Based Content Caching Process

As shown in Algorithm 1, the PPO algorithm optimizes content caching decisions by iteratively adjusting the policy parameters by using a clipped objective function and advantage estimation to maximize cumulative rewards. The process starts with initializing the actor network’s policy parameters θ and $\theta_{\mathrm{old}}$ to ensure consistent learning. The critic network is initialized with parameters ϕ to evaluate state values. Key hyperparameters, including learning rate α, discount factor γ, and clipping parameter ϵ, are set to guide the training process. This setup forms the foundation for the algorithm’s ability to adapt and optimize caching strategies dynamically. Then, the environment is reset to its initial state in each episode. At each time step, the agent observes the current state ($s_{t}^{ca}$), selects an action $a_{t}^{ca}$ based on the current policy θ, executes the action, and receives a reward $r_{t}$.

The critic network, parameterized by ϕ, is trained by using gradient descent to minimize the loss function

(20) $$\begin{array}{c} L\left(\phi \right)=\sum_{t=1}^{T}{\left(\delta_{t}\right)}^{2}, \end{array}$$

where $\delta_{t}=r_{t}+\gamma V_{\phi }\left(s_{t+1}^{ca}\right)-V_{\phi }\left(s_{t}^{ca}\right)$. The generalized advantage estimator (GAE) is as shown in

(21) $$\begin{array}{c} A_{t}=\sum_{l=0}^{\infty }{\left(\gamma \lambda \right)}^{l}\delta_{t+l}. \end{array}$$

The actor network updates its policy parameters θ by maximizing a clipped objective function, ensuring stable updates. The clipped objective function is defined as follows:

(22) $$\begin{array}{c} J_{\mathrm{PPO}}^{\theta }\left(\theta \right)=\sum_{\left(s_{t},a_{t}\right)}\min\left(wA_{t},\mathrm{clip}\left(w,1-\epsilon ,1+\epsilon \right)A_{t}\right), \end{array}$$

where ϵ is a clip fraction. The policy ratio of the target policy to the behavior policy can be expressed as

(23) $$\begin{array}{c} w=\frac{\pi_{\theta }\left(a_{t}^{ca}|s_{t}^{ca}\right)}{\pi_{\theta_{\mathrm{old}}}\left(a_{t}^{ca}|s_{t}^{ca}\right)}. \end{array}$$

Periodically, the behavior policy parameters are synchronized with the current policy to maintain consistency. By iteratively repeating these steps, the PPO algorithm effectively learns optimal caching strategies, adapting to dynamic changes in the network environment and improving overall performance.
**Algorithm 1:** PPO-based content caching process
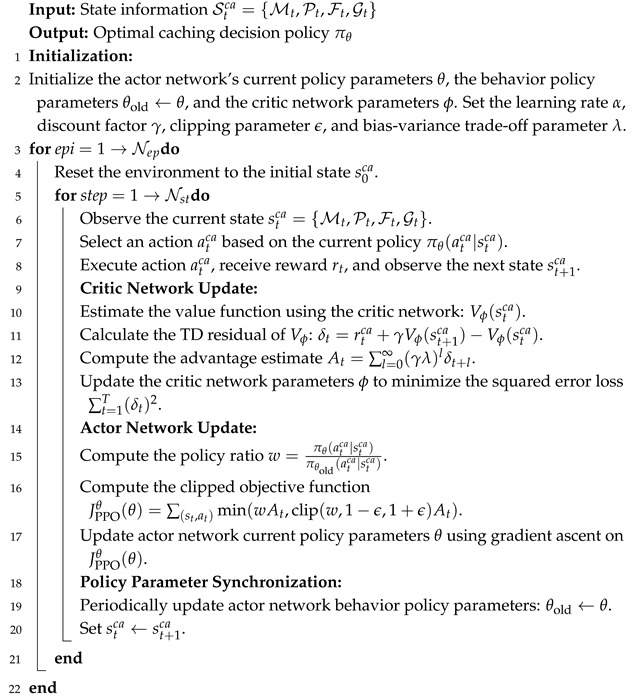


## 5. Offloading Decision, UAV Hovering Position, and STAR-RIS Resource Allocation

Once content caching has been solved by PPO, computing offloading decision α, UAV hovering position $r_{u}$, and STAR-RIS passive beamforming Θ can be further optimized by KKT and SCA iteratively.

(24a) $$\begin{array}{cc} P_{3}:\;\; & \min_{\begin{array}{c} \alpha ,r_{u},\Theta \end{array}}\sum_{m\in M}\sum_{k\in K}\left(\left(1-\sum_{j^{\prime }\in \left\{u,c\right\}}{X_{j^{\prime }}^{k}}^{*}\right)\left(E_{m}^{\mathrm{tr},\;k}+E_{m}^{com,k}\right)+\sum_{j^{\prime }\in \left\{u,c\right\}}{X_{j^{\prime }}^{k}}^{*}E_{m,j^{\prime }}^{cache,k}\right)\\ s.t.\;\; & \left(C_{1}\right)-\left(C_{5}\right),\left(C_{7}\right),\left(C_{9}\right), \end{array}$$

(24b) $$\begin{array}{cc} \; & \sum_{k\in K}{X_{j^{\prime }}^{k}}^{*}S^{k}\leq O_{j^{\prime }},\forall k,\forall j^{\prime }, \end{array}$$

(24c) $$\begin{array}{cc} \; & {X_{j^{\prime }}^{k}}^{*}\in \left\{0,1\right\},\forall k,\forall j^{\prime }. \end{array}$$

### 5.1. Offloading Decision

Given $X=X^{*}$, $r_{u}=r_{u}^{0}$, and $\Theta =\Theta^{0}$, problem (15) is rewritten as

(25a) $$\begin{array}{cc} \min_{\begin{array}{c} \alpha \end{array}}= & \sum_{m\in M}\sum_{k\in K}\left(\left(1-\sum_{j^{\prime }\in \left\{u,c\right\}}{X_{j^{\prime }}^{k}}^{*}\right)\left(\sum_{j^{\prime }\in \left\{u,c\right\}}P_{m}\frac{\alpha_{m,j^{\prime }}^{k}L_{k}}{R_{m,j^{\prime }}^{0}}+\sum_{j\in \{m,u,c\}}\kappa_{j}\alpha_{m,j}^{k}\omega_{k}f_{j}^{2}\right)\right)\\ s.t.\;\; & \left(\text{15a}\right),\;\left(\text{15b}\right),\;\left(\text{15g}\right), \end{array}$$

(25b) $$\begin{array}{cc} \; & {T_{m}^{k}}^{0}\leq T_{m}^{k,\max},\forall m,\forall k, \end{array}$$

where in $R_{m,j^{\prime }}^{0}$ and ${T_{m}^{k}}^{0}$, $r_{u}=r_{u}^{0}$ and $\Theta =\Theta^{0}$.

Consider the non-convexity of problem (25) under the given $X=X^{*}$, $r_{u}^{0}$, and $\Theta^{0}$. A proximal quadratic regularization term, i.e., $\sum_{m\in M}\sum_{k\in K}\xi {\alpha_{m,j}^{k}}^{2}$, is added to (25a) to overcome the issue, where ξ is a positive scalar parameter. The regularized problem is equivalent to the original one in (25) as ξ→ 0. Therefore, $E_{prox}^{tot}\left(X^{*},\alpha ,r_{u}^{0},\Theta^{0}\right)$ can be written as

(26) $$\begin{array}{cc} \; & E_{prox}^{tot}\left(X^{*},\alpha ,r_{u}^{0},\Theta^{0}\right)=\sum_{m\in M}\sum_{k\in K}\left(\left(1-\sum_{j^{\prime }\in \left\{u,c\right\}}{X_{j^{\prime }}^{k}}^{*}\right)\right.\\ \; & \left.\left(\sum_{j^{\prime }\in \left\{u,c\right\}}P_{m}\frac{\alpha_{m,j^{\prime }}^{k}L_{k}}{R_{m,j^{\prime }}^{0}}+\sum_{j\in \{m,u,c\}}\left(\kappa_{j}\alpha_{m,j}^{k}\omega_{k}f_{j}^{2}+\xi {\alpha_{m,j}^{k}}^{2}\right)\right)\right). \end{array}$$

Hence, problem (25) is rewritten as

(27a) $$\begin{array}{cc} \min_{\begin{array}{c} \alpha \end{array}}\; & E_{prox}^{tot}\left(X^{*},\alpha ,r_{u}^{0},\Theta^{0}\right) \end{array}$$

(27b) $$\begin{array}{cc} s.t.\;\; & \left(\text{15a}\right),\;(\text{15b}),\;(\text{15g}),\;(\text{25b}). \end{array}$$

According to (27), $E_{prox}^{tot}\left(X^{*},\alpha ,r_{u}^{0},\Theta^{0}\right)$ is a function of α, and its Hessian matrix is semi-positive definite. Given $X^{*}$, $r_{u}^{0}$, and $\Theta^{0}$, the optimal task offloading decision vector of problem (27) is convex and can be obtained by using the KKT conditions. The Lagrange function of problem (27), denoted by L(α,ε,χ,ζ,ξ), is written as (28), where ε, χ, ζ, and ξ are the nonnegative Lagrangian multipliers associated with constraints (15a), (15b), (15g), and (25b), respectively.

(28) $$\begin{array}{cc} L(\alpha ,\varepsilon ,\chi ,\zeta ,\xi )= & \sum_{m\in M}\sum_{k\in K}\left\{\left(1-\sum_{j^{\prime }\in \left\{u,c\right\}}{X_{j^{\prime }}^{k}}^{*}\right)\left(\sum_{j^{\prime }\in \left\{u,c\right\}}P_{m}\frac{\alpha_{m,j^{\prime }}^{k}L_{k}}{R_{m,j^{\prime }}^{0}}+\sum_{j\in \{m,u,c\}}\left(\xi {\alpha_{m,j}^{k}}^{2}\right.\right.\right.\\ \; & \left.\left.\left.+\kappa_{j}\alpha_{m,j}^{k}\omega_{k}f_{j}^{2}\right)\right)\right\}+\varepsilon_{m}\left(\alpha_{m,j}^{k}-1\right)+\chi_{m}\left(\sum_{j\in \{m,u,c\}}\left(\alpha_{m,j}^{k}-1\right)\right)\\ \; & +\zeta_{m}\left(\sum_{k\in K}\alpha_{m,j}^{k}\omega_{k}-f_{j}\right)+\xi_{m}\left({T_{m}^{k}}^{0}-T_{m}^{k,\max}\right). \end{array}$$

According to the KKT conditions, the optimal task offloading decision vector $\alpha^{*}$ is given by

(29) $$\alpha^{*}=\underset{\left\{\tilde{\alpha },\tilde{\varepsilon },\tilde{\chi },\tilde{\zeta },\tilde{\xi }\right\}}{\arg\min}\;E_{prox}^{tot}\left(X^{*},\alpha ,r_{u}^{0},\Theta^{0}\right).$$

If $\left\{\tilde{\alpha },\tilde{\varepsilon },\tilde{\chi },\tilde{\zeta },\tilde{\xi }\right\}$ is any point in the feasible solution set ${℧}_{\alpha }$ that satisfies the KKT conditions (30), by solving (30), the feasible solution set ${℧}_{\alpha }$ can be derived. The optimal task offloading decisions can be obtained, as given in (29).

(30a) $$\begin{array}{cc} \; & \frac{\partial L}{\partial \alpha_{m,j}^{k}}{|}_{\alpha_{m,j}^{k}={\tilde{\alpha }}_{m,j}^{k},\varepsilon_{m}={\tilde{\varepsilon }}_{m},\chi_{m}={\tilde{\chi }}_{m},\zeta_{m}={\tilde{\zeta }}_{i},\xi_{m}={\tilde{\xi }}_{i}}=0,\forall m,\forall k,\forall j, \end{array}$$

(30b) $$\begin{array}{cc} \; & 0\leq {\tilde{\alpha }}_{m,j}^{k}\leq 1,\forall m,\forall k,\forall j, \end{array}$$

(30c) $$\begin{array}{cc} \; & \sum_{j\in \{m,u,c\}}{\tilde{\alpha }}_{m,j}^{k}=1,\forall m,\forall k,\forall j, \end{array}$$

(30d) $$\begin{array}{cc} \; & \sum_{k\in K}{\tilde{\alpha }}_{m,j}^{k}\omega_{k}\leq f_{j},\forall m,\forall k,\forall j, \end{array}$$

(30e) $$\begin{array}{cc} \; & {\tilde{T_{m}^{k}}}^{0}\leq T_{m}^{k,\max},\forall m,\forall k,\forall j, \end{array}$$

(30f) $$\begin{array}{cc} \; & {\tilde{\varepsilon }}_{m}\left({\tilde{\alpha }}_{m,j}^{k}-1\right)=0,\forall m,\forall k,\forall j, \end{array}$$

(30g) $$\begin{array}{cc} \; & {\tilde{\chi }}_{m}\left(\sum_{j\in \{m,u,c\}}\left({\tilde{\alpha }}_{m,j}^{k}-1\right)\right)=0,\forall m,\forall k,\forall j, \end{array}$$

(30h) $$\begin{array}{cc} \; & {\tilde{\zeta }}_{m}\left(\sum_{k\in K}{\tilde{\alpha }}_{m,j}^{k}\omega_{k}-f_{j}\right)=0,\forall m,\forall k,\forall j, \end{array}$$

(30i) $$\begin{array}{cc} \; & {\tilde{\xi }}_{m}\left({\tilde{T_{m}^{k}}}^{0}-T_{m}^{k,\max}\right)=0,\forall m,\forall k,\forall j, \end{array}$$

(30j) $$\begin{array}{cc} \; & {\tilde{\varepsilon }}_{m}\geq 0,\forall m, \end{array}$$

(30k) $$\begin{array}{cc} \; & {\tilde{\chi }}_{m}\geq 0,\forall m, \end{array}$$

(30l) $$\begin{array}{cc} \; & {\tilde{\zeta }}_{m}\geq 0,\forall m, \end{array}$$

(30m) $$\begin{array}{cc} \; & {\tilde{\xi }}_{m}\geq 0,\forall m. \end{array}$$

### 5.2. UAV Hovering Position

Given $X=X^{*}$, $\alpha =\alpha^{*}$, and $\Theta =\Theta^{0}$, problem (15) is rewritten as

(31a) $$\begin{array}{cc} \min_{\begin{array}{c} r_{u} \end{array}}= & \sum_{m\in M}\sum_{k\in K}\left(\left(1-\sum_{j^{\prime }\in \left\{u,c\right\}}{X_{j^{\prime }}^{k}}^{*}\right)\sum_{j^{\prime }\in \left\{u,c\right\}}P_{m}\frac{\alpha_{m,j^{\prime }}^{k}L_{k}}{R_{m,j^{\prime }}^{0}}\right)\\ s.t.\;\; & \left(\text{15e}\right), \end{array}$$

(31b) $$\begin{array}{cc} \; & {T_{m}^{k}}^{0}\leq T_{m}^{k,\max},\forall m,\forall k, \end{array}$$

where in $R_{m,j^{\prime }}^{0}$ and ${T_{m}^{k}}^{0}$, $\Theta =\Theta^{0}$.

We adopt alternative optimization techniques and decompose the UAV hovering position problem into two steps: (1) Given $y_{u}=y_{u}^{0}$, problem (31) optimizes $x_{u}$, and we adopt SCA and the dual decomposition method to obtain an optimal solution, denoted by $x_{u}^{*}$. (2) Given $x_{u}=x_{u}^{*}$, problem (31) optimizes $y_{u}$, and we also adopt SCA and the dual decomposition method to obtain an optimal solution, denoted by $y_{u}^{*}$.

Given $y_{u}=y_{u}^{0}$, problem (31) can be rewritten as

(32a) $$\begin{array}{cc} \min_{\begin{array}{c} x_{u} \end{array}}= & \sum_{m\in M}\sum_{k\in K}\left(\left(1-\sum_{j^{\prime }\in \left\{u,c\right\}}{X_{j^{\prime }}^{k}}^{*}\right)\sum_{j^{\prime }\in \left\{u,c\right\}}P_{m}\frac{\alpha_{m,j^{\prime }}^{k}L_{k}}{R_{m,j^{\prime }}^{0}}\right) \end{array}$$

(32b) $$\begin{array}{cc} s.t.\;\; & x_{u}\leq x_{u}^{\max}, \end{array}$$

(32c) $$\begin{array}{cc} \; & {T_{m}^{k}}^{0}\leq T_{m}^{k,\max},\forall m,\forall k, \end{array}$$

where in $R_{m,j^{\prime }}^{0}$ and ${T_{m}^{k}}^{0}$, $y_{u}=y_{u}^{0}$ and $\Theta =\Theta^{0}$.

The non-convexity of objective (32a) arises from the hovering position $x_{u}$ in $r_{u}$ in $\log_{2}\left(1+\gamma_{m,j^{\prime }}^{0}\right)$. Considering that $R_{m,j^{\prime }}^{0}$ is replaced by their approximate variables, denoted by ${\tilde{R}}_{m,j^{\prime }}^{0}$, to transform (32) into a convex function, problem (32) can be reformulated as

(33a) $$\begin{array}{cc} \min_{\begin{array}{c} x_{u} \end{array}} & \sum_{m\in M}\sum_{k\in K}\left(\left(1-\sum_{j^{\prime }\in \left\{u,c\right\}}{X_{j^{\prime }}^{k}}^{*}\right)\sum_{j^{\prime }\in \left\{u,c\right\}}P_{m}\frac{\alpha_{m,j^{\prime }}^{k}L_{k}}{{\tilde{R}}_{m,j^{\prime }}^{0}}\right)\\ s.t.\;\; & \left(\text{32b}\right), \end{array}$$

(33b) $$\begin{array}{cc} \; & {\tilde{T_{m}^{k}}}^{0}\leq T_{m}^{k,\max},\forall m,\forall k, \end{array}$$

(33c) $$\begin{array}{cc} \; & B_{m,j^{\prime }}\log_{2}\left(1+\gamma_{m,j^{\prime }}^{0}\right)\geq {\tilde{R}}_{m,j^{\prime }}^{0},\forall m,\forall j^{\prime }. \end{array}$$

To address the non-convexity of (33c), the SCA method is used to derive a near-optimal solution. We first convexify $\log_{2}\left(1+\gamma_{m,j^{\prime }}^{0}\right)$ by utilizing a logarithmic approximation as follows:

(34) $$\log_{2}\left(1+\gamma_{m,j^{\prime }}^{0}\right)\geq \frac{\delta_{m,j^{\prime }}\ln\gamma_{m,j^{\prime }}^{0}+\lambda_{m,j^{\prime }}}{\ln2},$$

which is tight when $\gamma_{m,j^{\prime }}^{0}$ = ${\tilde{\gamma }}_{m,j^{\prime }}^{0}$. $\delta_{i,u}$ and $\lambda_{m,j^{\prime }}$ are two approximation constants about $\gamma_{m,j^{\prime }}^{0}$, defined as follows:

(35) $$\delta_{m,j^{\prime }}=\frac{{\tilde{\gamma }}_{m,j^{\prime }}^{0}}{1+{\tilde{\gamma }}_{m,j^{\prime }}^{0}};$$

(36) $$\lambda_{m,j^{\prime }}=\ln\left(1+{\tilde{\gamma }}_{m,j^{\prime }}^{0}\right)-\frac{{\tilde{\gamma }}_{m,j^{\prime }}^{0}}{1+{\tilde{\gamma }}_{m,j^{\prime }}^{0}}\ln{\tilde{\gamma }}_{m,j^{\prime }}^{0}.$$

Let $x_{u}={\tilde{x}}_{u}$. Then, constraint (33c) can be approximated by its concave lower bound, as given by

(37) $$R_{m,j^{\prime }}\geq B_{m,j^{\prime }}\frac{\delta_{m,j^{\prime }}\ln\gamma_{m,j^{\prime }}^{0}\left({\tilde{x}}_{u}\right)+\lambda_{m,j^{\prime }}}{\ln2}.$$

Therefore, problem (33) can be approximated by a convex problem, as given by

(38a) $$\begin{array}{cc} \min_{\begin{array}{c} x_{u} \end{array}} & \sum_{m\in M}\sum_{k\in K}\left(\left(1-\sum_{j^{\prime }\in \left\{u,c\right\}}{X_{j^{\prime }}^{k}}^{*}\right)\sum_{j^{\prime }\in \left\{u,c\right\}}P_{m}\frac{\alpha_{m,j^{\prime }}^{k}L_{k}}{{\tilde{R}}_{m,j^{\prime }}^{0}}\right)\\ s.t.\;\; & \left(\text{32b}\right),\;(\text{33b}), \end{array}$$

(38b) $$\begin{array}{cc} \; & B_{m,j^{\prime }}\frac{\delta_{m,j^{\prime }}\ln\gamma_{m,j^{\prime }}^{0}\left({\tilde{x}}_{u}\right)+\lambda_{m,j^{\prime }}}{\ln2}\geq {\tilde{R}}_{m,j^{\prime }}^{0},\forall m,\forall j^{\prime }. \end{array}$$

The optimal hovering position $x_{u}^{*}$ in $r_{u}^{*}$ of UAV *u* can be readily solved by using CVX. We utilize the Lagrangian dual approach for better efficiency. The Lagrangian function $L(x_{u},\varsigma ,\iota ,\omega )$ is given in (39), where ς, ι, and ω are the Lagrangian multipliers associated with the constraints of problem (38).

(39) $$\begin{array}{cc} \; & L\left(x_{u},\varsigma ,\iota ,\omega \right)=\sum_{m\in M}\sum_{k\in K}\left(\left(1-\sum_{j^{\prime }\in \left\{u,c\right\}}{X_{j^{\prime }}^{k}}^{*}\right)\sum_{j^{\prime }\in \left\{u,c\right\}}P_{m}\frac{\alpha_{m,j^{\prime }}^{k}L_{k}}{{\tilde{R}}_{m,j^{\prime }}^{0}}\right)+\varsigma_{m}\left(x_{u}-x_{u}^{max}\right)\\ \; & +\iota_{m}\left({\tilde{T_{m}^{k}}}^{0}-T_{m}^{k,\max}\right)+\omega_{m}\left({\tilde{R}}_{m,j^{\prime }}^{0}-B_{m,j^{\prime }}\frac{\delta_{m,j^{\prime }}\ln\gamma_{m,j^{\prime }}^{0}\left({\tilde{x}}_{u}\right)+\lambda_{m,j^{\prime }}}{\ln2}\right). \end{array}$$

The Lagrangian dual function of (39) can be given by

(40) $$\begin{array}{c} D\left(\varsigma ,\iota ,\omega \right)=\min_{\begin{array}{c} x_{u} \end{array}}L\left(x_{u},\varsigma ,\iota ,\omega \right). \end{array}$$

With the convexity of problem (38), the optimal solutions of both the original problem (38) and the dual problem (40) satisfy the KKT conditions. By solving $\frac{\partial L\left(x_{u},\varsigma ,\iota ,\omega \right)}{\partial x_{u}}=0$, the optimal solution $x_{u}^{*}$ is derived. The optimal hovering position $r_{u}^{*}=\left(x_{u}^{*},y_{u},h_{u}\right)$, where $x_{u}^{*}$ can be obtained as

(41) $$\begin{array}{c} x_{u}^{*}=\underset{x_{u}}{\arg\min}L\left(x_{u},\varsigma ,\iota ,\omega \right). \end{array}$$

The Lagrangian multipliers ς, ι, and ω are given by

(42) $$\begin{array}{cc} \varsigma_{m}\left[k+1\right]= & {\left[\varsigma_{m}\left[k\right]+{△}_{\varsigma }\left[k\right]\left(x_{u}-x_{u}^{max}\right)\right]}^{+}; \end{array}$$

(43) $$\begin{array}{cc} \iota_{m}\left[k+1\right]= & {\left[\iota_{i}\left[k\right]+{△}_{\iota }\left[k\right]\left({\tilde{T_{m}^{k}}}^{0}-T_{m}^{k,\max}\right)\right]}^{+}; \end{array}$$

(44) $$\begin{array}{cc} \omega_{m}\left[k+1\right]= & {\left[\omega_{m}\left[k\right]+{△}_{\omega }\left[k\right]\left({\tilde{R}}_{m,j^{\prime }}^{0}-B_{m,j^{\prime }}\frac{\delta_{m,j^{\prime }}\ln\gamma_{m,j^{\prime }}^{0}\left({\tilde{x}}_{u}\right)+\lambda_{m,j^{\prime }}}{\ln2}\right)\right]}^{+}, \end{array}$$

where ${△}_{1}\left[k\right]=({△}_{\varsigma }\left[k\right],{△}_{\iota }\left[k\right],{△}_{\omega }\left[k\right]$ is the step size vector to update the Lagrangian multipliers ς,ι, and ω in the *k*-th iteration, respectively. ${△}_{1}$ is also updated per iteration. Without loss of generality, we assume that each element in ${△}_{1}$ has the same step size in an iteration.

According to problem (41), the optimal solution $x_{u}^{*}$ is derived. Given $x_{u}=x_{u}^{*}$, problem (31) is non-convex, we also adopt SCA and the dual decomposition method to obtain an optimal solution, denoted by $y_{u}^{*}$. Therefore, the optimal hovering position can be given by

(45) $$\begin{array}{c} r_{u}^{*}=\left(x_{u}^{*},y_{u}^{*},h_{u}\right). \end{array}$$

### 5.3. STAR-RIS Resource Allocation

Given $X=X^{*}$, $\alpha =\alpha^{*}$, and $r_{u}=r_{u}^{*}$, problem (15) can be rewritten as

(46a) $$\begin{array}{cc} \min_{\begin{array}{c} \Theta \end{array}}\; & \sum_{m\in M}\sum_{k\in K}\left(\left(1-{X_{c}^{k}}^{*}\right)P_{m}\frac{\alpha_{m,c}^{k}L_{k}}{{\tilde{R}}_{m,c}^{\prime }}\right)\\ s.t.\;\; & \left(\text{15c}\right),\;(\text{15d}), \end{array}$$

(46b) $$\begin{array}{cc} \; & {T_{m}^{k}}^{\prime }\leq T_{m}^{k,\max},\forall m,\forall k, \end{array}$$

where in $R_{m,c}^{\prime }$ and ${T_{m}^{k}}^{\prime }$, $X=X^{*}$, $\alpha =\alpha^{*}$, and $r_{u}=r_{u}^{*}$.

The non-convexity of both objective (46a) and constraint (46b) arises from Θ in $\log_{2}\left(1+\gamma_{m,c}^{\prime }\right)$ for the given $X^{*}$, $\alpha^{*}$, and $r_{u}^{*}$. Similarly, an approximate variable ${\tilde{\gamma }}_{m,c}^{\prime }$ replaces $\gamma_{m,c}^{\prime }$ to overcome its non-convexity. Problem (46) is rewritten as

(47a) $$\begin{array}{cc} \min_{\begin{array}{c} \Theta \end{array}}\; & \sum_{m\in M}\sum_{k\in K}\left(\left(1-{X_{c}^{k}}^{*}\right)P_{m}\frac{\alpha_{m,c}^{k}L_{k}}{{\tilde{R}}_{m,c}^{\prime }}\right)\\ s.t.\;\; & \left(\text{15c}\right),\;(\text{15d}), \end{array}$$

(47b) $$\begin{array}{cc} \; & {\tilde{T_{m}^{k}}}^{\prime }\leq T_{m}^{k,\max},\forall m,\forall k, \end{array}$$

(47c) $$\begin{array}{cc} \; & \gamma_{m,c}^{\prime }\geq {\tilde{\gamma }}_{m,c}^{\prime },\forall m. \end{array}$$

Being part of $\gamma_{m,c}^{\prime }$, ${\left|G_{s,c}^{H}\Theta_{a}H_{m,s}\right|}^{2}$ can be rewritten as

(48) $${\left|G_{s,c}^{H}\Theta_{a}H_{m,s}\right|}^{2}={\left|\nu_{a}^{H}\phi_{s,c}^{H}H_{m,s}\right|}^{2}=\nu_{a}^{H}\Upsilon \nu_{a},$$

where $\nu_{a}^{H}$, $\phi_{s,c}^{H}$, and Υ can be written as

(49) $$\begin{array}{cc} \nu_{a}^{H}= & \left[\sqrt{\beta_{\left(1,1\right)}^{a}}e^{j\theta_{\left(1,1\right)}^{a}},\sqrt{\beta_{\left(1,2\right)}^{a}}e^{j\theta_{\left(1,2\right)}^{a}},\ldots ,\right.\\ \; & \left.\sqrt{\beta_{\left(m_{c},m_{r}\right)}^{a}}e^{j\theta_{\left(m_{c},m_{r}\right)}^{a}},\ldots ,\sqrt{\beta_{\left(M_{c},M_{r}\right)}^{a}}e^{j\theta_{\left(M_{c},M_{r}\right)}^{a}}\right]; \end{array}$$

(50) $$\begin{array}{cc} \phi_{s,c}^{H}= & \mathrm{diag}\left[g_{(1,1),c},g_{(1,2),c},\ldots ,g_{(m_{c},m_{r}),c},\ldots ,g_{(M_{c},M_{r}),c}\right]; \end{array}$$

(51) $$\begin{array}{cc} \Upsilon = & \phi_{s,c}^{H}H_{m,s}H_{m,s}^{H}\phi_{s,c}. \end{array}$$

As a result, constraints (15c) and (15d) are replaced by the following constraint:

(52) $$\sum_{a\in \left\{r,t\right\}}\nu_{a}^{2}\left[(m_{c},m_{r})\right]\leq 1,$$

where $\nu_{a}\left[(m_{c},m_{r})\right]$ is the $\left(m_{c},m_{r}\right)$-th element of $\nu_{a}^{H}$. Clearly, (52) is convex.

To tackle non-convex constraint (47c), the SCA method is adopted. Specifically, since $\nu_{a}^{H}\Upsilon \nu_{a}$ is convex about $\nu_{a}$, its lower bound can be derived as follows:

(53) νaHΥνa≥−νanHΥνan+2ReνaHΥνan,

where $\nu_{a}^{\left(n\right)}$ is obtained in the previous iteration. Then, constraint (47c) can be replaced by the following constraint:

(54) −νanHΥνan+2ReνaHΥνan≥γ˜m,c′σc2Pm,

which is linear. As a result, problem (47) is rewritten as

(55) $$\begin{array}{cc} \min_{\begin{array}{c} \nu_{a} \end{array}} & \sum_{m\in M}\sum_{k\in K}\left(\left(1-{X_{c}^{k}}^{*}\right)P_{m}\frac{\alpha_{m,c}^{k}L_{k}}{{\tilde{R}}_{m,c}^{\prime }}\right)\\ s.t.\;\; & \left(\text{47b}\right),\;(52),\;(54). \end{array}$$

Although convex problem (55) can be solved, e.g., by using the CVX toolbox, we utilize the Lagrangian dual approach to improve the computational efficiency. The Lagrangian function L(ν,ϱ,ϖ,ρ) is given in (56), where ϱ, ϖ, and ρ are the Lagrangian multipliers associated with the three constraints of problem (55).

(56) L(ν,ϱ,ϖ,ρ)=∑k∈K1−Xck*Pmαm,ckLkR˜m,c′+ϱmTmk˜′−Tmk,max+ϖm∑a∈r,tνa2(mc,mr)−1+ρmγ˜m,c′σc2Pm+νanHΥνan−2ReνaHΥνan.

The Lagrangian dual function is written as

(57) $$D\left(\varrho ,\varpi ,\rho \right)=\min_{\begin{array}{c} \nu \end{array}}L\left(\nu ,\varrho ,\varpi ,\rho \right).$$

With the convexity of problem (55), the optimal solutions of both the original problem (55) and problem (57) satisfy the KKT conditions. By solving $\frac{\partial L\left(\nu ,\varrho ,\varpi ,\rho \right)}{\partial \nu_{a}}=0$, the optimal solution $\nu_{a}^{*}$ is derived. The optimal $\nu_{a}^{*}$ is obtained as

(58) $$\nu_{a}^{*}=\underset{\nu_{a}}{\arg\min}L\left(\nu ,\varrho ,\varpi ,\rho \right).$$

The Lagrangian multipliers ϱ,ϖ, and ρ are given by

(59) $$\begin{array}{cc} \varrho_{m}\left[k+1\right]= & {\left\{\varrho_{m}\left[k\right]+{△}_{\varrho }\left[k\right]\left[{\tilde{T_{m}^{k}}}^{\prime }-T_{m}^{k,\max}\right]\right\}}^{+}; \end{array}$$

(60) $$\begin{array}{cc} \varpi_{m}\left[k+1\right]= & {\left\{\varpi_{m}\left[k\right]+{△}_{\varpi }\left[k\right]\left[\sum_{a\in \left\{r,t\right\}}\nu_{a}^{2}\left[(m_{c},m_{r})\right]-1\right]\right\}}^{+}; \end{array}$$

(61) ρmk+1=ρm[k]+△ρ[k]γ˜m,c′σc2Pm+νa(n)HΥνa(n)−2Re[νaHΥνa(n)]+,

where ${△}_{2}\left[k\right]={\left({△}_{\varrho }\left[k\right],{△}_{\varpi }\left[k\right],{△}_{\rho }\left[k\right]\right)}^{T}$ is the step size vector to update the Lagrangian multipliers ϱ, ϖ, and ρ in the *k*-th iteration, respectively. ${△}_{2}$ is updated in each iteration.

According to (49) and (58), the optimal transmission and reflection matrix $\Theta_{a}^{*}$ is obtained as

(62) $$\Theta_{a}^{*}=\mathrm{diag}{\left(\nu_{a}^{*}\right)}^{H}.$$

## 6. Convergence and Complexity Analysis

Algorithm 2 provides details of the DRL-SCA algorithm in its entirety. As a whole loop, the generated action $X^{*}$ by the PPO algorithm will be taken into (29), (38), and (55) as a given parameter for computing offloading decision, UAV hovering position, and STAR-RIS passive beamforming optimization. Then, utilizing block coordinate descent (BCD) to iterative optimize computing offloading decision $\alpha^{*}$, UAV hovering position $r_{u}=\left(x_{u}^{*},y_{u}^{*},h_{u}\right)$, and STAR-RIS passive beamforming Θ. Each iteration’s solution serves as the input of feasible points for the subsequent one. Therefore, $\alpha^{*}$, $r_{u}^{*}$, and $\Theta^{*}$ will become a new state for the PPO agent in the next time slot.
**Algorithm 2:** DRL-SCA-based caching decision, offloading decision, hovering position of the UAV, and passive beamforming of STAR-RIS for UAV-based WSNs
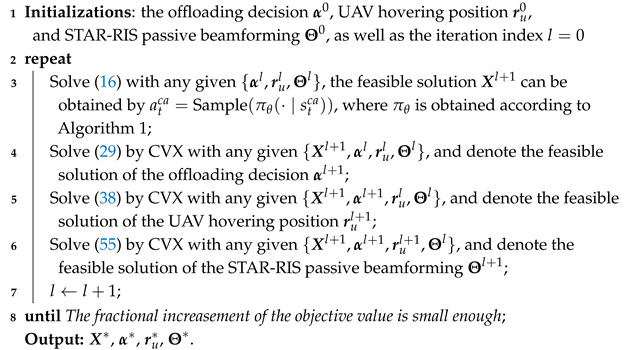


Therefore, the change of action will eventually lead to the transition of state, which drives the agent to learn to improve the operation objective-related reward. Even if the system finds it hard to obtain an analytical prediction due to the environment complexity and embedded SCA, deep reinforcement learning can still pave the way to figure out a feasible solution in such a complicated dynamic environment.

**Lemma 1.**  *At least in finite iterations, Algorithm 2 can converge to a local suboptimal solution.*

**Proof.**  The initial problem (15) is decomposed into four subproblems and addressed iteratively by using the BCD method. Specifically, subproblems (16), (29), (38), and (55) are optimized in an alternating sequence to acquire a suboptimal solution. The solution obtained in each iteration is subsequently used as the feasible input for the following iteration.

Let $\eta (X^{l},\alpha^{l},r_{u}^{l},\Theta^{l})$ represent the value of the original objective function (15) obtained during the *l*-th iteration.

The DRL algorithm generates an improved solution $X^{l+1}$ in Step 3 that meets the condition

(63) $$\begin{array}{c} \eta \left(X^{l},\alpha^{l},r_{u}^{l},\Theta^{l}\right)\overset{\left(a\right)}{\geq }\eta \left(X^{l+1},\alpha^{l},r_{u}^{l},\Theta^{l}\right), \end{array}$$

where (a) follows the inherent nature of learning approaches that always tend to seek a better reward defined in (19).

In Step 4, the suboptimal solution for offloading decision $\alpha^{l+1}$ can be obtained by solving (29), where (b) follows its convexity. In Step 5, the suboptimal solution for UAV hovering position $r_{u}^{l+1}$ can be obtained by solving (38), where (c) follows its convexity at the given feasible point due to constraints (32b), (33b), and (38b), and it can be optimally solved due to its convexity. In Step 6, the suboptimal solution for STAR-RIS passive beamforming $\Theta^{l+1}$ can be obtained by solving (55), where (d) follows its convexity at the given feasible point due to constraints (47b), (52), and (54), and it can be optimally solved due to its convexity.

(64) $$\begin{array}{cc} \eta (X^{l+1},\alpha^{l},r_{u}^{l},\Theta^{l})\; & \overset{\left(b\right)}{\geq }\eta \left(X^{l+1},\alpha^{l+1},r_{u}^{l},\Theta^{l}\right)\\ \; & \overset{\left(c\right)}{\geq }\eta \left(X^{l+1},\alpha^{l+1},r_{u}^{l+1},\Theta^{l}\right)\\ \; & \overset{\left(d\right)}{\geq }\eta \left(X^{l+1},\alpha^{l+1},r_{u}^{l+1},\Theta^{l+1}\right). \end{array}$$

The inequality of Equation (64) induces that subproblems (29), (38), and (55) with regard to energy consumption are always nonincreasing after each iteration. It is observed that the objective function does not increase with each iteration. Due to the constraints, the minimum achievable energy consumption is bounded below by a finite value. Consequently, Algorithm 2 is assured to converge to at least a locally suboptimal solution within a finite number of iterations. □

## 7. Performance Evaluation and Discussion

In this section, we present numerical results to evaluate the effectiveness of the proposed energy-efficient aerial STAR-RIS-aided computing offloading and content caching framework for WSNs. The simulation scenario and parameter settings are initially outlined, followed by an in-depth discussion of the simulation results.

### 7.1. Simulation Scenario and Parameter Settings

In this section, we evaluate the performance of the proposed energy-efficient aerial STAR-RIS-aided computing offloading and content caching framework through numerical experiments. The simulation setup is detailed below.

We assume that the reference locations of the ground edge cloud (*c*), the UAV (*u*), and the STAR-RIS are positioned at (0,20,0) meters, $\left(x_{u}^{*},y_{u}^{*},20\right)$ meters, and $\left(x_{u}^{*},y_{u}^{*},20\right)$ meters, respectively. Additionally, the four ground sensors are fixed at (−40,40,0) meters, (−40,−40,0) meters, (40,40,0) meters, and (40,−40,0) meters, representing a typical distributed sensor network configuration [37]. The detailed simulation parameters are summarized in Table 2 [38]. For example, the data size to be transmitted is set to $L_{k}=6\times 10^{8}$ bits, representing a typical high-volume data transmission scenario in UAV-based WSNs. Moreover, we modeled data transmission as periodic, assuming a consistent flow of information typical of high-demand applications [39]. The maximum hovering range of the UAV is constrained within 40 m ($x_{u}^{max},y_{u}^{max}=40$ m), while the communication bandwidth $B_{m,j^{\prime }}$ is set to 3.2 MHz, ensuring sufficient transmission capacity for the tasks. $w_{k}$ represents the 600 r/bit computing resources required to complete task *k*. $M_{c}\times M_{r}$ indicates that the STAR-RIS consists of nine passive units.

For the caching DRL algorithm, detailed parameter settings are provided to ensure stability and convergence of the learning process: Different learning rates α are evaluated (e.g., 0.0001, 0.0003, and 0.0005) to assess their impact on convergence speed and stability. A learning rate of 0.0003 is found to provide the best balance between performance and robustness. The clipping parameter ϵ is set to 0.2. This parameter constrains policy updates, ensuring stable and gradual improvements during training. The discount factor γ is fixed at 0.99, which balances immediate rewards and long-term benefits, crucial to achieving consistent performance across episodes. These parameter settings were chosen to optimize the performance of the PPO algorithm within the DRL-SCA framework and ensure the reliable operation of the proposed model under dynamic conditions [40,41].

Moreover, to demonstrate the advantages of the proposed framework, we evaluate its performance in multiple existing comparative scenarios, including (1) full offloading, (2) fixed positions, (3) no STAR-RIS, and (4) no caching [30,42]. These existing comparative setups provide insights into the effectiveness of the proposed joint optimization framework in improving energy efficiency and overall system performance. All simulation scenarios are detailed below:

(1) Proposed policy: We minimize the total energy consumption by jointly optimizing the content caching decision, offloading decision, the hovering position of the UAV, and the STAR-RIS transmission and reflection coefficient matrix in the STAR-RIS-aided UAV system.
(2) Fixed-position policy: The fixed-position policy is to set the UAV position at a fixed position without dynamic adjustment. The reason for setting this comparison strategy is to analyze the effectiveness and advantages of optimizing the UAV hovering position.
(3) Full offloading policy: The full offloading policy mainly offloads all request tasks sent by users to the UAV or cloud for independent processing. The reason for setting this comparison strategy is to analyze the effectiveness and advantages of the partial offloading strategy.
(4) No-STAR-RIS policy: The no-STAR-RIS policy is to directly transmit the task request sent by the user to the edge cloud without using the STAR-RIS transmission and reflection coefficient matrix for transmission. The reason for setting this comparison strategy is to analyze the effectiveness and advantages of the STAR-RIS transmission and reflection coefficient matrix.
(5) No-caching policy: The no–caching strategy consists of not to using the cache service capability on the UAV or edge cloud and not caching the requested content sent by the user in advance. Therefore, under this strategy, the user directly uses the partial offloading strategy to process the task locally or in the UAV or edge cloud. The reason for setting this comparison strategy is to analyze the effectiveness and advantages of the content caching strategy.

### 7.2. The Discussion of the Simulation Results

This section evaluates and discusses the performance of the proposed DRL-SCA algorithm in the energy-efficient aerial STAR-RIS-aided computing offloading and content caching framework by comparing it with various benchmark methods across diverse scenarios.

**Figure 6:**
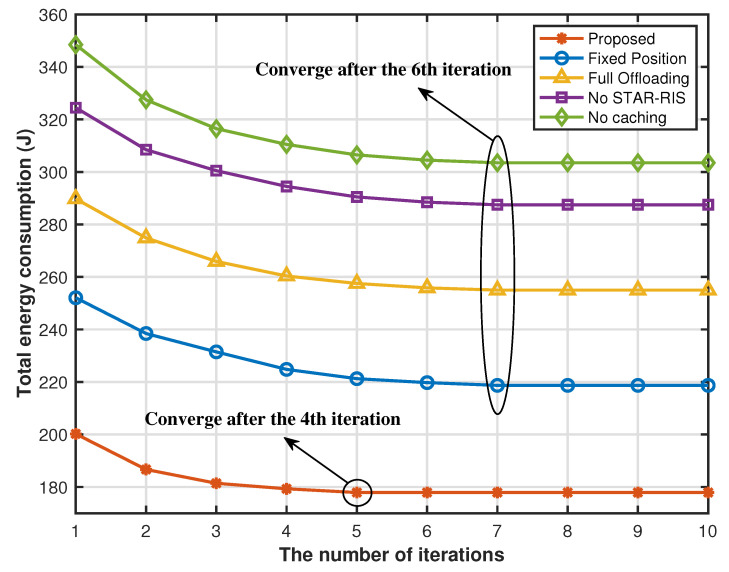
Energy consumption versus the number of iterations.

Figure 6 plots the total energy consumption of the system versus the number of iterations. We see that the proposed method can ensure that the total energy consumption of the system converges to the optimal value after only serval iterations, confirming that the optimal caching strategy, partial offloading strategy, the hovering position of the UAV, and the STAR-RIS transmission and reflection matrix are always available. Moreover, the convergence speed of the four benchmark schemes is slightly slower than that of the proposed scheme. Finally, this figure can verify that the energy consumption of the proposed STAR-RIS-aided computing offloading and content caching framework for UAV systems is less than that of other benchmark schemes.

**Figure 7:**
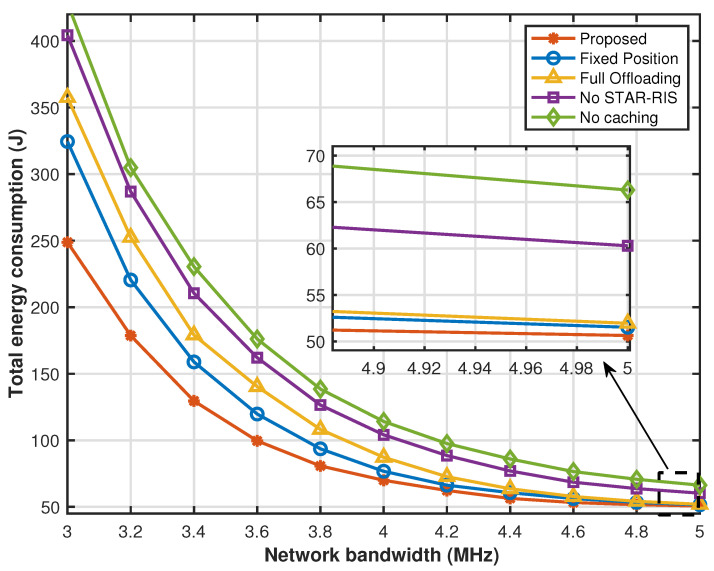
Energy consumption versus network bandwidth.

Figure 7 shows the total energy consumption of the system versus the network bandwidth. The results show that the total energy consumption of the five schemes decreases with the increase in network bandwidth. The reason is that the increase in network bandwidth for offloading improves the transmission rate between sensors and the UAV, as well as the transmission rate among the sensors, the STAR-RIS, and the edge cloud, reducing the transmission delay and energy consumption. At a lower network bandwidth, the proposed caching strategy optimization, UAV hovering position optimization, partial offloading optimization, and STAR-RIS transmission reflection coefficient matrix significantly reduce energy consumption compared with other comparison strategies. However, as the network bandwidth increases, it helps to increase the task transmission rate, making the impact of network bandwidth in system energy consumption dominant. The system energy consumption of other benchmark solutions gradually approaches that of the proposed solution, but through the proposed joint optimization policy, our system performance is always optimal.

**Figure 8:**
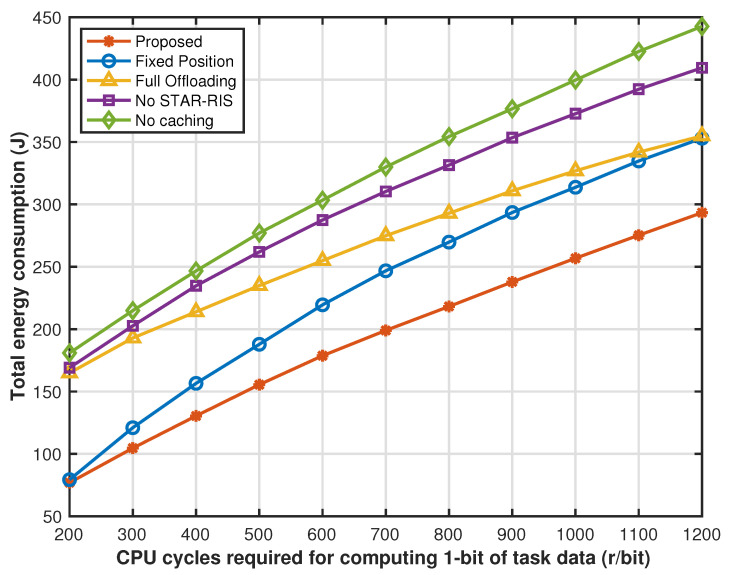
Energy consumption versus CPU cycles required for computing 1 bit of task data.

Figure 8 shows that as the CPU cycles required for computing 1 bit of task data increase, the system consumes more computing resources when calculating tasks of the same size, thereby increasing computing energy consumption. However, when the computing power of drones and local sensors is limited, more tasks are offloaded to the edge cloud for processing, which increases transmission energy consumption. At the same time, as the number of offloaded tasks increases, network resources become increasingly limited. Under the condition of limited network resources, the cache strategy optimization, drone hovering position optimization, and STAR-RIS transmission reflection coefficient matrix optimization strategies we proposed can significantly reduce system transmission energy consumption compared with other comparison strategies. Therefore, the system energy consumption gap between the proposed solution and other benchmark solutions will gradually widen. However, as network resources become increasingly limited, the network energy consumption under full offloading is only affected by computing resources, resulting in an increase in computing energy consumption, and the transmission energy consumption does not change much. Therefore, the system energy consumption of full offloading solutions gradually approaches that of the proposed partial offloading solution.

**Figure 9:**
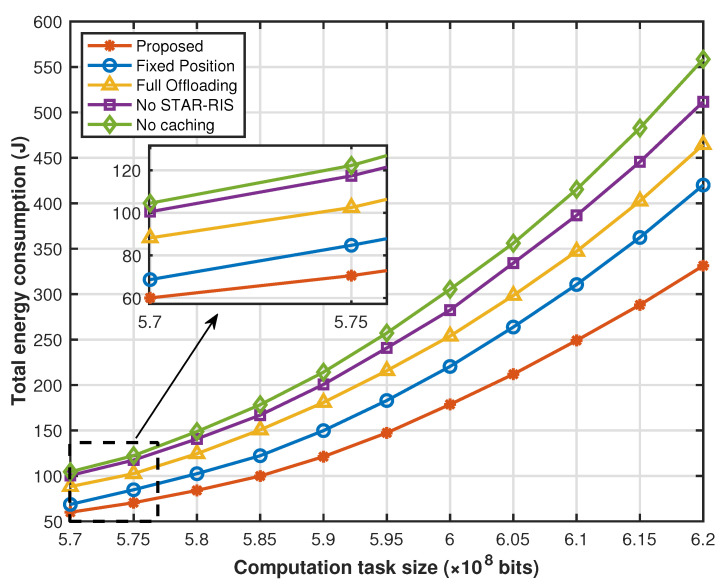
Energy consumption versus computation task size.

Figure 9 evaluates the total energy consumption of the proposed scheme and other benchmark schemes for various computation task sizes. First, the increase in computation task sizes leads to an increase in the total energy consumption of the system, because as the computation task sizes increase, transmission energy consumption gradually increases, and the computing energy consumption for processing unit tasks also increases. When local sensors and drones are unable to handle tasks due to their computation capability limitations, more computation tasks have to be offloaded to the distant edge cloud for processing. This not only increases computing energy consumption but also, due to the limited resources of UAVs and local sensors, offloading tasks to a distant cloud for processing, greatly increasing transmission energy consumption. Furthermore, the advantage of the proposed scheme over other benchmark schemes in the total energy consumption is marginal when we set the computation task size to small, while the advantage becomes increasingly substantial upon increasing the task size.

**Figure 10:**
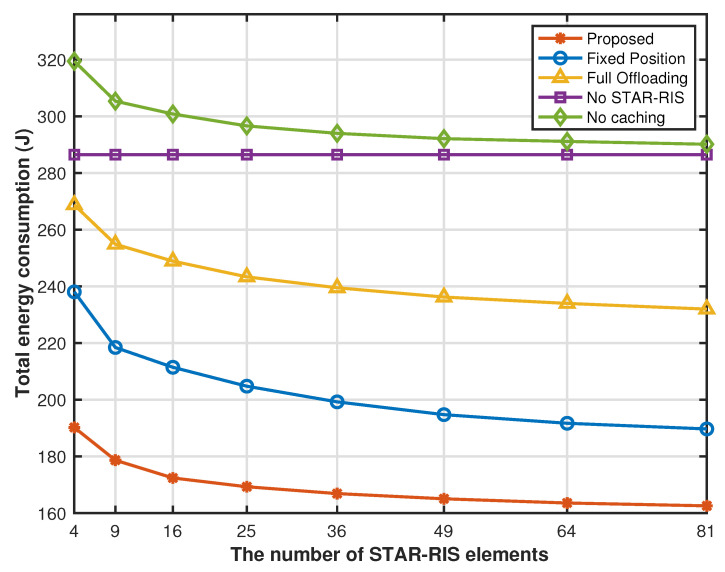
Energy consumption versus number of elements.

Figure 10 shows that the system energy consumption of all STAR-RIS-related schemes decreases significantly as the number of STAR-RIS elements increases. This is because STAR-RIS elements can provide more channel gain and effectively reduce transmission energy consumption. However, the no-STAR-RIS policy directly transmits some task requests sent by the sensor to the edge cloud without using the STAR-RIS transmission reflection coefficient matrix for transmission. Therefore, as the number of STAR-RIS elements increases, the system energy consumption under this strategy remains unchanged. At a smaller number of STAR-RIS elements, the energy consumption of the proposed cache strategy optimization, drone hovering position optimization, partial offloading optimization, and STAR-RIS transmission reflection coefficient matrix are significantly reduced compared with other comparison strategies. However, as the number of STAR-RIS elements increases, this provides more channel gain, which greatly reduces the transmission energy consumption of the system offloading tasks to a more distant cloud for processing. Therefore, the gap between the system energy consumption of other benchmark solutions and the energy consumption of the proposed solution gradually narrows, but through the proposed joint optimization strategy, our system performance is always in the optimal state.

**Figure 11:**
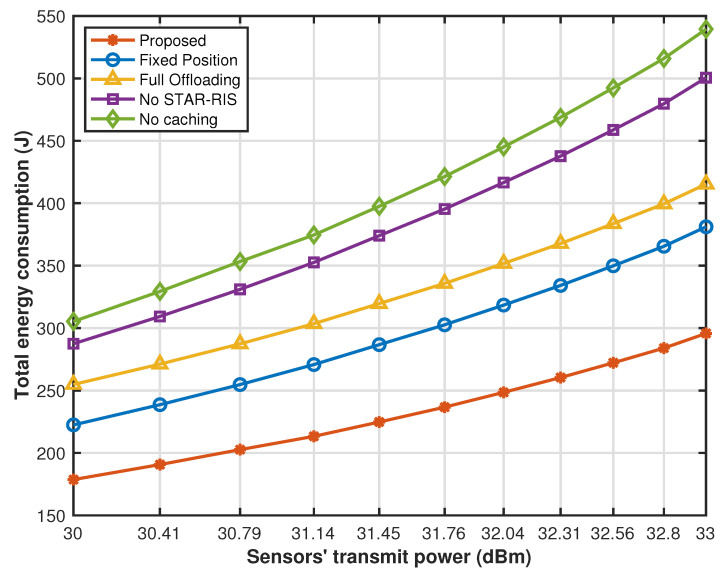
Energy consumption versus sensors’ transmit power.

Figure 11 illustrates that the system energy consumption of all schemes increases significantly with the increase in the sensors’ transmit power. This is because as the sensor’s transmission power increases, the transmission delay gradually decreases, but the transmission energy consumption is proportional to the sensor’s power. Therefore, when the sensor’s offloading ratio remains unchanged, the overall energy consumption of the system gradually increases. At smaller sensor’s transmit power, the energy consumption of the proposed cache strategy optimization, drone hovering position optimization, partial offloading optimization, and STAR-RIS transmission reflection coefficient matrix is significantly reduced compared with other comparison strategies. However, as the sensor’s transmission power increases, it greatly increases the transmission energy consumption of the system offloading tasks to the edge cloud or UAV for processing. Therefore, the gap between the system energy consumption of other benchmark solutions and the energy consumption of the proposed solution gradually narrows, but through the proposed joint optimization strategy, our system performance is always in the optimal state.

**Figure 12:**
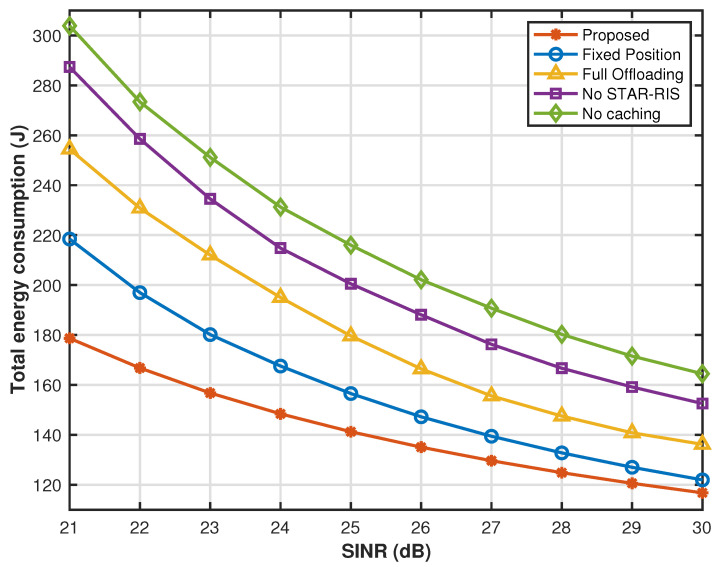
Energy consumption versus SINR.

We define the SINR as $\gamma_{n,n^{\prime }}=\frac{P_{n}{\left|h_{n,n^{\prime }}\right|}^{2}}{\sigma_{n,n^{\prime }}^{2}}$. It can be seen from Figure 12 that at greater SINR, the signal strength is relative to the noise. That is to say, the signal transmission quality will be better, thus improving transmission efficiency, which helps to reduce energy consumption during the transmission process. At a smaller SINR, the energy consumption of the proposed caching strategy optimization, UAV hovering position optimization, partial offloading optimization, and STAR-RIS transmission reflection coefficient matrix is significantly reduced compared with other comparison strategies. However, as the SINR increases, this provides a better channel state, which greatly reduces the transmission energy consumption of the system offloading tasks to a more distant cloud for processing. Therefore, the gap between the system energy consumption of other benchmark solutions and the energy consumption of the proposed solution gradually narrows, but through the proposed joint optimization strategy, our system performance is always in the optimal state.

**Figure 13:**
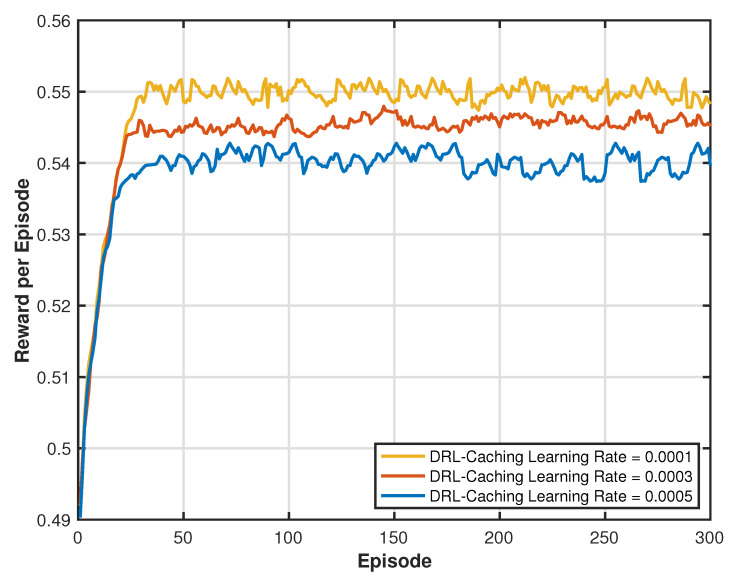
Convergence of average weighted reward sum for various caching DRL learning rates.

Figure 13 illustrates the convergence of the average weighted reward per episode for the DRL caching agent at varying learning rates. As seen in the figure, it is evident that the DRL caching agent converges rapidly at all learning rates and achieves optimal performance at a learning rate of 0.0003. A higher learning rate results in the current Q-value having more influence than the prior Q-value, leading to faster updates. However, excessively high learning rates may destabilize the learning process and hinder convergence by over-adjusting to recent rewards. Therefore, selecting an appropriate learning rate is crucial to balancing the learning speed and stability for optimal performance.

The above simulation results demonstrate the effectiveness of the proposed DRL-SCA algorithm in optimizing energy consumption in the aerial STAR-RIS-aided computing offloading and content caching framework. Across various scenarios, our joint optimization strategy consistently outperforms benchmark methods. Specifically, the results reveal that the proposed framework achieves faster convergence in energy optimization, as shown by the rapid decrease in energy consumption over iterations. The analysis highlights that key factors such as network bandwidth, computation task size, CPU cycles per bit, STAR-RIS element count, and sensors’ transmission power significantly impact the system’s energy performance. For instance, the proposed strategy exhibits substantial energy savings, particularly under conditions of limited network resources or high computational demand, owing to the efficient coordination of offloading and caching decisions, UAV hovering positions, and STAR-RIS passive beamforming. Additionally, the results demonstrate that higher STAR-RIS element counts and better SINR conditions further enhance energy efficiency by improving channel gain and transmission quality. The sensitivity analysis of DRL caching learning rates confirms the stability and rapid convergence of the DRL component, with optimal performance achieved at a learning rate of 0.0003. Overall, the results validate that the proposed framework achieves superior energy efficiency and scalability while maintaining robustness under dynamic conditions, showcasing its applicability in real-world UAV-based wireless sensor networks.

## 8. Conclusions

In this paper, the energy-efficient STAR-RIS-aided computing offloading and content caching framework is proposed in order to meet the service requirements of delay-sensitive tasks for UAV-based WSNs. Firstly, we formulated the system energy consumption minimization problem, aiming to jointly optimize content caching decisions, computing offloading decisions, UAV hovering positions, and STAR-RIS passive beamforming. Subsequently, to tackle the non-convex problem of system energy consumption minimization, we decomposed it into four subproblems and proposed a DRL-SCA algorithm for iterative optimization, achieving near-optimal solutions with low complexity. According to numerical results, the suggested framework significantly reduces the network energy consumption of the overall system in aerial STAR-RIS-aided WSNs, exhibiting a fast convergence rate.

In our future research, we plan to investigate the impact of various design parameters, including the power-to-weight ratio, to further optimize the overall system performance. We will explore the application of multi-agent proximal policy optimization (MAPPO) to enhance the content caching decisions, computing offloading decisions, UAV hovering positions, and STAR-RIS passive beamforming decision process by enabling collaborative optimization among multiple agents, further improving adaptability and the overall performance of the framework. To enhance real-world applicability, future work will focus on developing lightweight optimization models for key parameters and testing the framework under dynamic conditions, such as varying data loads, network congestion, and hardware constraints. Additionally, we will incorporate more complex real-world factors, including UAV mobility, multi-task coordination, and heterogeneous sensor networks, to further expand the framework’s applicability. Finally, we plan to explore advanced feature extraction and training techniques to improve the scalability of MAPPO algorithms for large-scale and complex network environments.

## Figures and Tables

**Table 1:** Summary of important notation.

Symbol	Definition
$M_{c}$	Total number of elements in each column of STAR-RIS
$M_{r}$	Total number of elements in each row of STAR-RIS
$r_{j}$	Location of node *j*
$r_{\left(m_{c},m_{r}\right)}$	Location of the $\left(m_{c},m_{r}\right)$-th STAR-RIS element
$h_{m,u}$	Channel gain from sensor *m* to UAV *u*
$h_{m,(m_{c},m_{r})}$	Channel gain from sensor *m* to the $\left(m_{c},m_{r}\right)$-th STAR-RIS element
$g_{(m_{c},m_{r}),c}$	Channel gain from the $\left(m_{c},m_{r}\right)$-th STAR-RIS element to edge cloud *c*
*P*	Transmit power
*B*	Spectrum bandwidth
*R*	Transmission rate
$f_{j}$	Computing capability (CPU cycles per second) of node *j*
α	Task offloading ratio
$\beta_{\left(m_{c},m_{r}\right)}^{a}$	Amplitude coefficients of transmitting and reflecting vectors ($a\in \left\{r,t\right\}$)
$\theta_{\left(m_{c},m_{r}\right)}^{a}$	The phase shift angle vectors of transmitting and reflecting elements ($a\in \left\{r,t\right\}$)
$k_{j}$	Effective switched capability of node *j*
$L_{k}$	The requested data size of task *k* (uplink)
$\omega_{k}$	Number of CPU cycles for computing 1-bit task *k*

**Table 2:** Simulation parameters.

Notation	Simulation Value	Notation	Simulation Value
$\kappa_{j}$	$1\times 10^{-28}$	$P_{m}$	30 dBm
$f_{c}$	$1\times 10^{10}$ r/s	$B_{m,j^{\prime }}$	3.2 MHz
$f_{u}$	$40\times 10^{7}$ r/s	$L_{k}$	$6\times 10^{8}$ bit
$f_{m}$	$2\times 10^{8}$ r/s	$\omega_{k}$	600 r/bit
$T_{m}^{k,\max}$	0.1 s	$M_{c}$×$M_{r}$	9
$g_{0}$	−30 dB	$x_{u}^{\max}$	40 m
$\sigma_{n,n^{\prime }}^{2}$	−174 dBm	$y_{u}^{\max}$	40 m

## Data Availability

The data analyzed during the current study are available from the corresponding author upon reasonable request.

## References

[1] Wang N.C., Chen Y.L., Huang Y.F., Huang L.C., Wang T.Y., Chuang H.Y. Energy Efficient Geocasting Based on Q-Learning for Wireless Sensor Networks. Proceedings of the 2019 International Conference on Machine Learning and Cybernetics (ICMLC).

[2] Akyildiz I.F., Melodia T., Chowdhury K.R. (2008). Wireless Multimedia Sensor Networks: Applications and Testbeds. Proc. IEEE.

[3] Shen J., Wang A., Wang C., Hung P.C.K., Lai C.F. (2017). An Efficient Centroid-Based Routing Protocol for Energy Management in WSN-Assisted IoT. IEEE Access.

[4] Ji J., Zhu K., Yi C., Niyato D. (2021). Energy Consumption Minimization in UAV-Assisted Mobile-Edge Computing Systems: Joint Resource Allocation and Trajectory Design. IEEE Internet Things J..

[5] Liao Z., Yin G., Tang X., Liu P. (2024). A Cooperative Community-Based Framework for Service Caching and Task Offloading in Multi-Access Edge Computing. IEEE Trans. Netw. Serv. Manag..

[6] Hao Y., Chen M., Hu L., Hossain M.S., Ghoneim A. (2018). Energy Efficient Task Caching and Offloading for Mobile Edge Computing. IEEE Access.

[7] Zhang K., Gui X., Ren D., Li D. (2021). Energy–Latency Tradeoff for Computation Offloading in UAV-Assisted Multiaccess Edge Computing System. IEEE Internet Things J..

[8] Hu X., Wong K.K., Yang K., Zheng Z. (2019). UAV-Assisted Relaying and Edge Computing: Scheduling and Trajectory Optimization. IEEE Trans. Wireless Commun..

[9] Zhang T., Wang Y., Liu Y., Xu W., Nallanathan A. (2020). Cache-Enabling UAV Communications: Network Deployment and Resource Allocation. IEEE Trans. Wireless Commun..

[10] Li M., Cheng N., Gao J., Wang Y., Zhao L., Shen X. (2020). Energy-Efficient UAV-Assisted Mobile Edge Computing: Resource Allocation and Trajectory Optimization. IEEE Trans. Veh. Technol..

[11] Gao X., Zhai L. (2024). Service Experience Oriented Cooperative Computing in Cache-Enabled UAVs Assisted MEC Networks. IEEE Trans. Mob. Comput..

[12] Zhong L., Yang S., Song K., Wang M., Jiang K., Muntean G.M. (2024). MDC2: An Integrated Communication and Computing Framework to Optimize Edge-Assisted Caching for Improved Multimedia Services in UAV-Based IoT Networks. IEEE Internet Things J..

[13] Guo H., Liu J. (2020). UAV-Enhanced Intelligent Offloading for Internet of Things at the Edge. IEEE Trans. Ind. Inform..

[14] Wu C., You C., Liu Y., Gu X., Cai Y. (2022). Channel Estimation for STAR-RIS-Aided Wireless Communication. IEEE Commun. Lett..

[15] Liu Z., Li Z., Wen M., Gong Y., Wu Y.C. (2023). STAR-RIS-Aided Mobile Edge Computing: Computation Rate Maximization With Binary Amplitude Coefficients. IEEE Trans. Commun..

[16] Yang S., Xie C., Lyu W., Ning B., Zhang Z., Yuen C. (2024). Near-Field Channel Estimation for Extremely Large-Scale Reconfigurable Intelligent Surface (XL-RIS)-Aided Wideband mmWave Systems. IEEE J. Sel. Areas Commun..

[17] Mozaffari M., Saad W., Bennis M., Nam Y.H., Debbah M. (2019). A Tutorial on UAVs for Wireless Networks: Applications, Challenges, and Open Problems. IEEE Commun. Surv. Tut..

[18] Qin X., Song Z., Hou T., Yu W., Wang J., Sun X. (2023). Joint Resource Allocation and Configuration Design for STAR-RIS-Enhanced Wireless-Powered MEC. IEEE Trans. Commun..

[19] Yu S., Langar R., Fu X., Wang L., Han Z. (2018). Computation Offloading With Data Caching Enhancement for Mobile Edge Computing. IEEE Trans. Veh. Technol..

[20] Wang J., Liu K., Pan J. (2020). Online UAV-Mounted Edge Server Dispatching for Mobile-to-Mobile Edge Computing. IEEE Internet Things J..

[21] Zhao Y., Liu C., Hu X., He J., Peng M., Ng D.W.K., Quek T.Q. (2024). Joint Content Caching, Service Placement and Task Offloading in UAV-Enabled Mobile Edge Computing Networks. IEEE J. Sel. Areas Commun..

[22] Huang J., Zhang M., Wan J., Chen Y., Zhang N. (2024). Joint Data Caching and Computation Offloading in UAV-assisted Internet of Vehicles via Federated Deep Reinforcement Learning. IEEE Trans. Veh. Technol..

[23] Aung P.S., Nguyen L.X., Tun Y.K., Han Z., Hong C.S. (2024). Aerial STAR-RIS Empowered MEC: A DRL Approach for Energy Minimization. IEEE Wireless Commun. Lett..

[24] Lyu W., Xiu Y., Yang S., Yeoh P.L., Li Y., Zhang Z. (2023). Weighted Sum Age of Information Minimization in Wireless Networks With Aerial IRS. IEEE Trans. Veh. Technol..

[25] Ren J., Yu G., He Y., Li G.Y. (2019). Collaborative Cloud and Edge Computing for Latency Minimization. IEEE Trans. Veh. Technol..

[26] Bi S., Huang L., Zhang Y.J.A. (2020). Joint Optimization of Service Caching Placement and Computation Offloading in Mobile Edge Computing Systems. IEEE Trans. Wireless Commun..

[27] Xiu Y., Zhao Y., Yang S., Xu M., Niyato D., Li Y., Wei N. (2024). Delay Minimization for Movable Antennas-Enabled Anti-Jamming Communications with Mobile Edge Computing. arXiv.

[28] Meng K., Wu Q., Xu J., Chen W., Feng Z., Schober R., Swindlehurst A.L. (2024). UAV-Enabled Integrated Sensing and Communication: Opportunities and Challenges. IEEE Wireless Commun..

[29] Wu H., Lyu F., Zhou C., Chen J., Wang L., Shen X. (2020). Optimal UAV Caching and Trajectory in Aerial-Assisted Vehicular Networks: A Learning-Based Approach. IEEE J. Sel. Areas Commun..

[30] Zhang Q., Zhao Y., Li H., Hou S., Song Z. (2022). Joint Optimization of STAR-RIS Assisted UAV Communication Systems. IEEE Wireless Commun. Lett..

[31] Wei Z., Cai Y., Sun Z., Ng D.W.K., Yuan J., Zhou M., Sun L. (2021). Sum-Rate Maximization for IRS-Assisted UAV OFDMA Communication Systems. IEEE Trans. Wireless Commun..

[32] Mu X., Liu Y., Guo L., Lin J., Schober R. (2022). Simultaneously Transmitting and Reflecting (STAR) RIS Aided Wireless Communications. IEEE Trans. Wireless Commun..

[33] Su Y., Pang X., Chen S., Jiang X., Zhao N., Yu F.R. (2022). Spectrum and Energy Efficiency Optimization in IRS-Assisted UAV Networks. IEEE Trans. Commun..

[34] Luo Y., Ding W., Zhang B. (2021). Optimization of Task Scheduling and Dynamic Service Strategy for Multi-UAV-Enabled Mobile-Edge Computing System. IEEE Trans. Cogn. Commun. Netw..

[35] Zhang Z., Chen J., Liu Y., Wu Q., He B., Yang L. (2022). On the Secrecy Design of STAR-RIS Assisted Uplink NOMA Networks. IEEE Trans. Wireless Commun..

[36] Zeng Y., Chen S., Cui Y., Yang J., Fu Y. (2023). Joint Resource Allocation and Trajectory Optimization in UAV-Enabled Wirelessly Powered MEC for Large Area. IEEE Internet Things J..

[37] Su Y., Pang X., Lu W., Zhao N., Wang X., Nallanathan A. (2023). Joint Location and Beamforming Optimization for STAR-RIS Aided NOMA-UAV Networks. IEEE Trans. Veh. Technol..

[38] Shnaiwer Y.N., Kaneko M. (2023). Minimizing IoT Energy Consumption by IRS-Aided UAV Mobile Edge Computing. IEEE Netw. Lett..

[39] Ma Z., Xiao M., Xiao Y., Pang Z., Poor H.V., Vucetic B. (2019). High-Reliability and Low-Latency Wireless Communication for Internet of Things: Challenges, Fundamentals, and Enabling Technologies. IEEE Internet Things J..

[40] Tang Q., Li B., Yang H.H., Li Y., He S., Yang K. (2024). Delay and Load Fairness Optimization with Queuing Model in Multi-UAV Assisted MEC: A Deep Reinforcement Learning Approach. IEEE Trans. Netw. Serv. Manag..

[41] Qin P., Fu Y., Zhang J., Geng S., Liu J., Zhao X. (2024). DRL-Based Resource Allocation and Trajectory Planning for NOMA-Enabled Multi-UAV Collaborative Caching 6G Network. IEEE Trans. Veh. Technol..

[42] Lin N., Bai L., Hawbani A., Guan Y., Mao C., Liu Z., Zhao L. (2024). Deep-Reinforcement-Learning-Based Computation Offloading for Servicing Dynamic Demand in Multi-UAV-Assisted IoT Network. IEEE Internet Things J..

